# Innate lymphoid cells and COVID-19 severity in SARS-CoV-2 infection

**DOI:** 10.7554/eLife.74681

**Published:** 2022-03-11

**Authors:** Noah J Silverstein, Yetao Wang, Zachary Manickas-Hill, Claudia Carbone, Ann Dauphin, Brittany P Boribong, Maggie Loiselle, Jameson Davis, Maureen M Leonard, Leticia Kuri-Cervantes, MGH COVID-19 Collection & Processing Team, Kendall Lavin-Parsons, Blair Parry, Brendan Lilley, Carl Lodenstein, Brenna McKaig, Nicole Charland, Hargun Khanna, Justin Margolin, Anna Gonye, Irena Gushterova, Tom Lasalle, Nihaarika Sharma, Brian C Russo, Maricarmen Rojas-Lopez, Moshe Sade-Feldman, Kasidet Manakongtreecheep, Jessica Tantivit, Molly Fisher Thomas, Betelihem A Abayneh, Patrick Allen, Diane Antille, Katrina Armstrong, Siobhan Boyce, Joan Braley, Karen Branch, Katherine Broderick, Julia Carney, Andrew Chan, Susan Davidson, Michael Dougan, David Drew, Ashley Elliman, Keith Flaherty, Jeanne Flannery, Pamela Forde, Elise Gettings, Amanda Griffin, Sheila Grimmel, Kathleen Grinke, Kathryn Hall, Meg Healy, Deborah Henault, Grace Holland, Chantal Kayitesi, Vlasta LaValle, Yuting Lu, Sarah Luthern, Jordan Marchewka, Brittani Martino, Roseann McNamara, Christian Nambu, Susan Nelson, Marjorie Noone, Christine Ommerborn, Lois Chris Pacheco, Nicole Phan, Falisha A Porto, Edward Ryan, Kathleen Selleck, Sue Slaughenhaupt, Kimberly Smith Sheppard, Elizabeth Suschana, Vivine Wilson, Galit Alter, Alejandro Balazs, Julia Bals, Max Barbash, Yannic Bartsch, Julie Boucau, Josh Chevalier, Fatema Chowdhury, Kevin Einkauf, Jon Fallon, Liz Fedirko, Kelsey Finn, Pilar Garcia-Broncano, Ciputra Hartana, Chenyang Jiang, Paulina Kaplonek, Marshall Karpell, Evan C Lam, Kristina Lefteri, Xiaodong Lian, Mathias Lichterfeld, Daniel Lingwood, Hang Liu, Jinqing Liu, Natasha Ly, Ashlin Michell, Ilan Millstrom, Noah Miranda, Claire O’Callaghan, Matthew Osborn, Shiv Pillai, Yelizaveta Rassadkina, Alexandra Reissis, Francis Ruzicka, Kyra Seiger, Libera Sessa, Christianne Sharr, Sally Shin, Nishant Singh, Weiwei Sun, Xiaoming Sun, Hannah Ticheli, Alicja Trocha-Piechocka, Daniel Worrall, Alex Zhu, George Daley, David Golan, Howard Heller, Arlene Sharpe, Nikolaus Jilg, Alex Rosenthal, Colline Wong, Nuala J Meyer, Michael R Betts, Jonathan Z Li, Bruce D Walker, Xu G Yu, Lael M Yonker, Jeremy Luban

**Affiliations:** Department of Emergency Medicine, Massachusetts General HospitalBostonUnited States; Department of Emergency Medicine, Massachusetts General HospitalBostonUnited States; Department of Emergency Medicine, Massachusetts General HospitalBostonUnited States; Department of Emergency Medicine, Massachusetts General HospitalBostonUnited States; Department of Emergency Medicine, Massachusetts General HospitalBostonUnited States; Department of Emergency Medicine, Massachusetts General HospitalBostonUnited States; Department of Emergency Medicine, Massachusetts General HospitalBostonUnited States; Department of Emergency Medicine, Massachusetts General HospitalBostonUnited States; Massachusetts General Hospital Cancer CenterBostonUnited States; Massachusetts General Hospital Cancer CenterBostonUnited States; Massachusetts General Hospital Cancer CenterBostonUnited States; Massachusetts General Hospital Cancer CenterBostonUnited States; Division of Infectious Diseases, Department of Medicine, Massachusetts General HospitalBostonUnited States; Division of Infectious Diseases, Department of Medicine, Massachusetts General HospitalBostonUnited States; Massachusetts General Hospital Center for Immunology and Inflammatory DiseasesBostonUnited States; Massachusetts General Hospital Center for Immunology and Inflammatory DiseasesBostonUnited States; Massachusetts General Hospital Center for Immunology and Inflammatory DiseasesBostonUnited States; Massachusetts General Hospital Center for Immunology and Inflammatory DiseasesBostonUnited States; Massachusetts General HospitalBostonUnited States; Massachusetts General HospitalBostonUnited States; Massachusetts General HospitalBostonUnited States; Massachusetts General HospitalBostonUnited States; Massachusetts General HospitalBostonUnited States; Massachusetts General HospitalBostonUnited States; Massachusetts General HospitalBostonUnited States; Massachusetts General HospitalBostonUnited States; Massachusetts General HospitalBostonUnited States; Massachusetts General HospitalBostonUnited States; Massachusetts General HospitalBostonUnited States; Massachusetts General HospitalBostonUnited States; Massachusetts General HospitalBostonUnited States; Massachusetts General HospitalBostonUnited States; Massachusetts General HospitalBostonUnited States; Massachusetts General HospitalBostonUnited States; Massachusetts General HospitalBostonUnited States; Massachusetts General HospitalBostonUnited States; Massachusetts General HospitalBostonUnited States; Massachusetts General HospitalBostonUnited States; Massachusetts General HospitalBostonUnited States; Massachusetts General HospitalBostonUnited States; Massachusetts General HospitalBostonUnited States; Massachusetts General HospitalBostonUnited States; Massachusetts General HospitalBostonUnited States; Massachusetts General HospitalBostonUnited States; Massachusetts General HospitalBostonUnited States; Massachusetts General HospitalBostonUnited States; Massachusetts General HospitalBostonUnited States; Massachusetts General HospitalBostonUnited States; Massachusetts General HospitalBostonUnited States; Massachusetts General HospitalBostonUnited States; Massachusetts General HospitalBostonUnited States; Ragon Institute of MGH, MIT and HarvardCambridgeUnited States; Massachusetts General HospitalBostonUnited States; Massachusetts General HospitalBostonUnited States; Massachusetts General HospitalBostonUnited States; Massachusetts General HospitalBostonUnited States; Massachusetts General HospitalBostonUnited States; Massachusetts General HospitalBostonUnited States; Massachusetts General HospitalBostonUnited States; Massachusetts General HospitalBostonUnited States; Massachusetts General HospitalBostonUnited States; Massachusetts General HospitalBostonUnited States; Massachusetts General HospitalBostonUnited States; Massachusetts General HospitalBostonUnited States; Ragon Institute of MGH, MIT and HarvardCambridgeUnited States; Ragon Institute of MGH, MIT and HarvardCambridgeUnited States; Ragon Institute of MGH, MIT and HarvardCambridgeUnited States; Ragon Institute of MGH, MIT and HarvardCambridgeUnited States; Ragon Institute of MGH, MIT and HarvardCambridgeUnited States; Ragon Institute of MGH, MIT and HarvardCambridgeUnited States; Ragon Institute of MGH, MIT and HarvardCambridgeUnited States; Ragon Institute of MGH, MIT and HarvardCambridgeUnited States; Ragon Institute of MGH, MIT and HarvardCambridgeUnited States; Ragon Institute of MGH, MIT and HarvardCambridgeUnited States; Ragon Institute of MGH, MIT and HarvardCambridgeUnited States; Ragon Institute of MGH, MIT and HarvardCambridgeUnited States; Ragon Institute of MGH, MIT and HarvardCambridgeUnited States; Ragon Institute of MGH, MIT and HarvardCambridgeUnited States; Ragon Institute of MGH, MIT and HarvardCambridgeUnited States; Ragon Institute of MGH, MIT and HarvardCambridgeUnited States; Ragon Institute of MGH, MIT and HarvardCambridgeUnited States; Ragon Institute of MGH, MIT and HarvardCambridgeUnited States; Ragon Institute of MGH, MIT and HarvardCambridgeUnited States; Ragon Institute of MGH, MIT and HarvardCambridgeUnited States; Ragon Institute of MGH, MIT and HarvardCambridgeUnited States; Ragon Institute of MGH, MIT and HarvardCambridgeUnited States; Ragon Institute of MGH, MIT and HarvardCambridgeUnited States; Ragon Institute of MGH, MIT and HarvardCambridgeUnited States; Ragon Institute of MGH, MIT and HarvardCambridgeUnited States; Ragon Institute of MGH, MIT and HarvardCambridgeUnited States; Ragon Institute of MGH, MIT and HarvardCambridgeUnited States; Ragon Institute of MGH, MIT and HarvardCambridgeUnited States; Ragon Institute of MGH, MIT and HarvardCambridgeUnited States; Ragon Institute of MGH, MIT and HarvardCambridgeUnited States; Ragon Institute of MGH, MIT and HarvardCambridgeUnited States; Ragon Institute of MGH, MIT and HarvardCambridgeUnited States; Ragon Institute of MGH, MIT and HarvardCambridgeUnited States; Ragon Institute of MGH, MIT and HarvardCambridgeUnited States; Ragon Institute of MGH, MIT and HarvardCambridgeUnited States; Ragon Institute of MGH, MIT and HarvardCambridgeUnited States; Ragon Institute of MGH, MIT and HarvardCambridgeUnited States; Ragon Institute of MGH, MIT and HarvardCambridgeUnited States; Ragon Institute of MGH, MIT and HarvardCambridgeUnited States; Ragon Institute of MGH, MIT and HarvardCambridgeUnited States; Ragon Institute of MGH, MIT and HarvardCambridgeUnited States; Ragon Institute of MGH, MIT and HarvardCambridgeUnited States; Ragon Institute of MGH, MIT and HarvardCambridgeUnited States; Ragon Institute of MGH, MIT and HarvardCambridgeUnited States; Ragon Institute of MGH, MIT and HarvardCambridgeUnited States; Harvard Medical SchoolBostonUnited States; Harvard Medical SchoolBostonUnited States; Harvard Medical SchoolBostonUnited States; Harvard Medical SchoolBostonUnited States; Brigham and Women’s HospitalBostonUnited States; Brigham and Women’s HospitalBostonUnited States; Brigham and Women’s HospitalBostonUnited States; 1 Program in Molecular Medicine, University of Massachusetts Medical School Worcester United States; 2 Medical Scientist Training Program, University of Massachusetts Medical School Worcester United States; 3 Massachusetts Consortium on Pathogen Readiness Boston United States; 4 Ragon Institute of MGH, MIT and Harvard Cambridge United States; 5 Massachusetts General Hospital, Mucosal Immunology and Biology Research Center Boston United States; 6 Massachusetts General Hospital, Department of Pediatrics Boston United States; 7 Harvard Medical School Boston United States; 8 Department of Microbiology, Perelman School of Medicine, University of Pennsylvania Philadelphia United States; 9 Institute for Immunology, Perelman School of Medicine, University of Pennsylvania Philadelphia United States; 10 Division of Pulmonary and Critical Care Medicine, Department of Medicine, University of Pennsylvania Perelman School of Medicine Philadelphia United States; 11 Department of Medicine, Brigham and Women’s Hospital Boston United States; 12 Howard Hughes Medical Institute Chevy Chase United States; 13 Department of Biology and Institute of Medical Engineering and Science, Massachusetts Institute of Technology Cambridge United States; 14 Department of Biochemistry and Molecular Biotechnology, University of Massachusetts Medical School Worcester United States; 15 Broad Institute of Harvard and MIT Cambridge United States; National and Kapodistrian University of Athens, Medical School Greece; Indian Institute of Science Education and Research (IISER) India

**Keywords:** SARS-CoV-2, COVID-19, MIS-C, innate lymphoid cells, disease tolerance, amphiregulin, Human

## Abstract

**Background:**

Risk of severe COVID-19 increases with age, is greater in males, and is associated with lymphopenia, but not with higher burden of SARS-CoV-2. It is unknown whether effects of age and sex on abundance of specific lymphoid subsets explain these correlations.

**Methods:**

Multiple regression was used to determine the relationship between abundance of specific blood lymphoid cell types, age, sex, requirement for hospitalization, duration of hospitalization, and elevation of blood markers of systemic inflammation, in adults hospitalized for severe COVID-19 (n = 40), treated for COVID-19 as outpatients (n = 51), and in uninfected controls (n = 86), as well as in children with COVID-19 (n = 19), recovering from COVID-19 (n = 14), MIS-C (n = 11), recovering from MIS-C (n = 7), and pediatric controls (n = 17).

**Results:**

This observational study found that the abundance of innate lymphoid cells (ILCs) decreases more than 7-fold over the human lifespan – T cell subsets decrease less than 2-fold – and is lower in males than in females. After accounting for effects of age and sex, ILCs, but not T cells, were lower in adults hospitalized with COVID-19, independent of lymphopenia. Among SARS-CoV-2-infected adults, the abundance of ILCs, but not of T cells, correlated inversely with odds and duration of hospitalization, and with severity of inflammation. ILCs were also uniquely decreased in pediatric COVID-19 and the numbers of these cells did not recover during follow-up. In contrast, children with MIS-C had depletion of both ILCs and T cells, and both cell types increased during follow-up. In both pediatric COVID-19 and MIS-C, ILC abundance correlated inversely with inflammation. Blood ILC mRNA and phenotype tracked closely with ILCs from lung. Importantly, blood ILCs produced amphiregulin, a protein implicated in disease tolerance and tissue homeostasis. Among controls, the percentage of ILCs that produced amphiregulin was higher in females than in males, and people hospitalized with COVID-19 had a lower percentage of ILCs that produced amphiregulin than did controls.

**Conclusions:**

These results suggest that, by promoting disease tolerance, homeostatic ILCs decrease morbidity and mortality associated with SARS-CoV-2 infection, and that lower ILC abundance contributes to increased COVID-19 severity with age and in males.

**Funding:**

This work was supported in part by the Massachusetts Consortium for Pathogen Readiness and NIH grants R37AI147868, R01AI148784, F30HD100110, 5K08HL143183.

## Introduction

The outcome of SARS-CoV-2 infection is highly variable with only a minority progressing to severe COVID-19, characterized by acute respiratory distress syndrome, multi-organ dysfunction, elevated inflammatory cytokines, lymphopenia, and other abnormalities of the immune system (Bonnet et al., 2021; Giamarellos-Bourboulis et al., 2020; Huang and Pranata, 2020; Kaneko et al., 2020; Kuri-Cervantes et al., 2020; Lucas et al., 2020; Mathew et al., 2020; Mudd et al., 2020; Zhou et al., 2020). The risk of severe COVID-19 and death in people infected with SARS-CoV-2 increases with age and is greater in men than in women (Alkhouli et al., 2020; Bunders and Altfeld, 2020; Gupta et al., 2021; Laxminarayan et al., 2020; Mauvais-Jarvis, 2020; O’Driscoll et al., 2021; Peckham et al., 2020; Richardson et al., 2020; Scully et al., 2020). These trends have been observed in people infected with SARS-CoV (Chen and Subbarao, 2007; Donnelly et al., 2003; Karlberg et al., 2004), or with MERS-CoV (Alghamdi et al., 2014), and in laboratory animals challenged with SARS-CoV or SARS-CoV-2 (Channappanavar et al., 2017; Leist et al., 2020). The mechanisms underlying these effects of age and sex on COVID-19 morbidity and mortality remain poorly understood.

The composition and function of the human immune system changes with age and exhibits sexual dimorphism (Darboe et al., 2020; Klein and Flanagan, 2016; Márquez et al., 2020; Patin et al., 2018; Solana et al., 2012), with consequences for survival of infection, response to vaccination, and susceptibility to autoimmune disease (Flanagan et al., 2017; Giefing-Kröll et al., 2015; Márquez et al., 2020; Mauvais-Jarvis, 2020; Patin et al., 2018; Piasecka et al., 2018). Better understanding of these effects might provide clues as to why the clinical outcome of SARS-CoV-2 infection is so variable, ranging from asymptomatic to lethal (Cevik et al., 2021; He et al., 2020; Jones et al., 2021; Lee et al., 2020; Lennon et al., 2020; Ra et al., 2021; Richardson et al., 2020; Yang et al., 2021).

Survival after infection with a pathogenic virus such as SARS-CoV-2 requires not only that the immune system control and eliminate the pathogen, but that disease tolerance mechanisms limit tissue damage caused by the pathogen or by host inflammatory responses (Ayres, 2020a; McCarville and Ayres, 2018; Medzhitov et al., 2012; Schneider and Ayres, 2008). Research with animal models has demonstrated that genetic and environmental factors can promote host fitness without directly inhibiting pathogen replication (Ayres, 2020a; Cumnock et al., 2018; Jhaveri et al., 2007; McCarville and Ayres, 2018; Medzhitov et al., 2012; Råberg et al., 2007; Sanchez et al., 2018; Schneider and Ayres, 2008; Wang et al., 2016). Although in most cases the underlying mechanism is unknown, some of these models suggest that subsets of innate lymphoid cells (ILCs) contribute to disease tolerance (Artis and Spits, 2015; Branzk et al., 2018; Califano et al., 2018; Diefenbach et al., 2020; McCarville and Ayres, 2018; Monticelli et al., 2015; Monticelli et al., 2011). Some ILC subsets produce the epidermal growth factor family member amphiregulin (AREG) that maintains the integrity of epithelial barriers in the lung and intestine (Branzk et al., 2018; Jamieson et al., 2013; Monticelli et al., 2015; Monticelli et al., 2011), and promotes tissue repair (Artis and Spits, 2015; Cherrier et al., 2018; Klose and Artis, 2016; Rak et al., 2016). In models of influenza infection in mice, homeostatic ILCs and exogenous AREG promote lung epithelial integrity, decrease disease severity, and increase survival, without decreasing pathogen burden (Califano et al., 2018; Jamieson et al., 2013; Monticelli et al., 2011).

Little is known about disease tolerance in the context of human infectious diseases. Interestingly, SARS-CoV-2 viral load does not reliably discriminate symptomatic from asymptomatic infection (Cevik et al., 2021; Jones et al., 2021; Lee et al., 2020; Lennon et al., 2021; Ra et al., 2021; Yang et al., 2021). This discrepancy between SARS-CoV-2 viral load and the severity of COVID-19 is especially pronounced in children, who rarely have severe COVID-19 (Bailey et al., 2021; Li et al., 2020; Lu et al., 2020; Poline et al., 2020), although viral load may be comparable to that in adults with severe COVID-19 (Heald-Sargent et al., 2020; LoTempio et al., 2021; Yonker et al., 2020). These observations suggest that age-dependent, disease tolerance mechanisms influence the severity of COVID-19. In mice, homeostatic ILCs decrease in abundance in the lung with increasing age, and lose their ability to maintain disease tolerance during influenza infection (D’Souza et al., 2019). Although the distribution of ILCs within human tissues differs from mice and is heterogeneous among individuals (Yudanin et al., 2019), human ILCs share many features with those in mice (Vivier et al., 2018) and therefore may perform similar roles in maintaining tissue homeostasis and disease tolerance.

ILCs in peripheral blood have been reported to be depleted in individuals with severe COVID-19 (García et al., 2020; Kuri-Cervantes et al., 2020), but it is difficult to determine the extent to which ILCs are decreased independently from the overall lymphopenia associated with COVID-19 (Chen et al., 2020; Huang et al., 2020; Huang and Pranata, 2020; Zhang et al., 2020; Zhao et al., 2020), or from changes in other blood cell lineages (Giamarellos-Bourboulis et al., 2020; Huang et al., 2020; Kuri-Cervantes et al., 2020; Lucas et al., 2020; Mathew et al., 2020; Mudd et al., 2020; Zheng et al., 2020). In addition, assessment of lymphoid cell abundance, in the context of a disease for which age and sex are risk factors for severity, is confounded by programmed differences in lymphocyte abundance with age and sex (Márquez et al., 2020; Patin et al., 2018). The goal of this study was to determine whether the abundance of any blood lymphoid cell population was altered in COVID-19, independent of age, sex, and global lymphopenia, and whether abundance of any lymphoid cell population correlated with clinical outcome in SARS-CoV-2 infection.

## Materials and methods

**Key resources table:** 

Reagent type (species) or resource	Designation	Source or reference	Identifiers	Additional information
Antibody	Anti-Human BDCA1 (mouse monoclonal)	Biolegend	Cat# 354,208	Clone: 201 A (FITC) (1:200 dilution)
Antibody	Anti-Human CD117 (mouse monoclonal)	Biolegend	Cat# 313,206	Clone: 104D2 (APC) (1:200 dilution)
Antibody	Anti-Human CD11c (mouse monoclonal)	Biolegend	Cat# 301,604	Clone: 3.9 (FITC) (1:200 dilution)
Antibody	Anti-Human CD123 (mouse monoclonal)	Biolegend	Cat# 306,014	Clone: 6H6 (FITC) (1:200 dilution)
Antibody	Anti-Human CD127 (mouse monoclonal)	Biolegend	Cat# 351,320	Clone: A019D5 (PE/Cyanine7) (1:200 dilution)
Antibody	Anti-Human CD14 (mouse monoclonal)	Biolegend	Cat# 325,604	Clone: HCD14 (FITC) (1:200 dilution)
Antibody	Anti-Human CD16 (mouse monoclonal)	Biolegend	Cat# 980,104	Clone: 3G8 (APC) (1:400 dilution)
Antibody	Anti-Human CD19 (mouse monoclonal)	Biolegend	Cat# 302,206	Clone: HIB19 (FITC) (1:200 dilution)
Antibody	Anti-Human CD1a (mouse monoclonal)	Biolegend	Cat# 300,104	Clone: HI149 (FITC) (1:200 dilution)
Antibody	Anti-Human CD20 (mouse monoclonal)	Biolegend	Cat# 302,304	Clone: 2H7a (FITC) (1:200 dilution)
Antibody	Anti-Human CD22 (mouse monoclonal)	Biolegend	Cat# 363,508	Clone: S-HCL-1 (FITC) (1:200 dilution)
Antibody	Anti-Human CD3 (mouse monoclonal)	Biolegend	Cat# 317,306	Clone: OKT3 (FITC) (1:200 dilution)
Antibody	Anti-Human CD34 (mouse monoclonal)	Biolegend	Cat# 343,504	Clone: 581 (FITC) (1:200 dilution)
Antibody	Anti-Human CD4 (mouse monoclonal)	Biolegend	Cat# 317,428	Clone: OKT4 (PerCP/Cyanine5.5) (1:200 dilution)
Antibody	Anti-Human CD4 (mouse monoclonal)	Biolegend	Cat# 317,408	Clone: OKT4 (FITC) (1:200 dilution)
Antibody	Anti-Human CD45 (mouse monoclonal)	BD	Cat# 560,178	Clone: 2D1 (APC/H7) (1:200 dilution)
Antibody	Anti-Human CD56 (mouse monoclonal)	Biolegend	Cat# 318,306	Clone: HCD56 (PE) (1:200 dilution)
Antibody	Anti-Human CD8 (mouse monoclonal)	Biolegend	Cat# 300,924	Clone: HIT8a (PerCP/Cyanine 5.5) (1:200 dilution)
Antibody	Anti-Human CRTH2 (rat monoclonal)	Biolegend	Cat# 350,116	Clone: BM16 (PerCP/Cyanine5.5) (1:200 dilution)
Antibody	Anti-Human FcεR1α (mouse monoclonal)	Biolegend	Cat# 334,608	Clone: AER-37 (FITC) (1:200 dilution)
Antibody	Anti-Human TBX21 (mouse monoclonal)	ebioscience	Cat# 25-5825-82	Clone: ebio4B10 (PE/Cyanine7) (1:200 dilution)
Antibody	Anti-Human TCRα/β (mouse monoclonal)	Biolegend	Cat# 306,706	Clone: IP26 (FITC) (1:200 dilution)
Antibody	Anti-Human TCRγ/δ (mouse monoclonal)	Biolegend	Cat# 331,208	Clone: B1 (FITC) (1:200 dilution)
Antibody	Anti-Human TCF7 (rabbit monoclonal)	Cell Signaling	Cat# 37,636 s	Clone: C63D9 (APC) (1:200 dilution)
Antibody	Anti-Human IL-13 (rat monoclonal)	Biolegend	Cat# 501,908	Clone: JES10-5A2 (APC) (1:200 dilution)
Antibody	Anti-Human AREG (mouse monoclonal)	ebioscience	Cat# 17-5370-42	Clone: AREG559
Antibody	mouse IgG1, k isotype control (mouse monoclonal)	Biolegend	Cat# 400,112	Clone: MOPC-21 (PE) (1:200 dilution)
Antibody	mouse IgG1, k isotype control (mouse monoclonal)	Biolegend	Cat# 400,120	Clone: MOPC-21 (APC) (1:200 dilution)
Antibody	Rabbit IgG, isotype control (rabbit monoclonal)	Cell Signaling	Cat# 3,452 S	(Alexa Fluor 647) (1:200 dilution)
Antibody	Rat IgG1, k isotype control (rat monoclonal)	Biolegend	Cat# 400,412	Clone: RTK2071 (APC) (1:200 dilution)
Biological Samples (*Homo sapiens*)	PBMCs	New York Biologics	https://www.newyorkbiologics.com/	
Biological Samples (*Homo sapiens*)	PBMCs	MassCPR	https://masscpr.hms.harvard.edu/	
Biological Samples (*Homo sapiens*)	PBMCs	MGH Pediatric COVID-19 Biorepository		
Commercial assay or kit	Cell stimulation cocktail	eBioscience	Cat# 00-4970-03	
Chemical compound, drug	Protein transport inhibitor	eBioscience	Cat# 00-4980-03	
Chemical compound, drug	TRIzol reagent	Invitrogen	Cat# 15596018	
Commercial assay or kit	AMPure XP beads	Beckman Culter	Cat# A63880	
Commercial assay or kit	ExoSAP-IT	Affymetrix	Cat# 78,200	
Commercial assay or kit	Live and Dead violet viability kit	Invitrogen	Cat# L-34963	
Commercial assay or kit	Foxp3 /Transcription Factor Staining Buffer Set	eBioscience	Cat# 00-5523-00	
Commercial assay or kit	HiScribe T7 High Yield RNA Synthesis Kit	NEB	Cat# E2040S	
Commercial assay or kit	NEBNext Ultra II Non directional Second Strand Synthesis Module	NEB	Cat# E6111L	
Software, algorithm	R computer software environment (version 4.0.2)	The R Foundation R Development Core Team, 2020	https://www.r-project.org/	
Software, algorithm	FlowJo	FlowJo, LLC	https://www.flowjo.com/	
Software, algorithm	tidyverse v1.3.1	Wickham et al., 2019	https://www.tidyverse.org	
Software, algorithm	ggplot2 v3.3.3	Wickham, 2016	https://ggplot2.tidyverse.org	
Software, algorithm	ggpubr v0.4.0	Kassambara, 2020	https://rpkgs.datanovia.com/ggpubr/	
Software, algorithm	ComplexHeatmap v2.4.3	Gu et al., 2016	https://github.com/jokergoo/ComplexHeatmap	
Software, algorithm	emmeans v1.6.0	Wieduwilt et al., 2020	https://CRAN.R-project.org/package=emmeans	
Software, algorithm	lme4 v1.1–27	Bates et al., 2015	https://cran.r-project.org/web/packages/lme4/index.html	
Software, algorithm	lmerTest v3.1–3	Kuznetsova et al., 2017	https://cran.r-project.org/web/packages/lmerTest/index.html	
Software, algorithm	DolphinNext RNA-Seq pipeline (Revision 4)	Yukselen et al., 2020	https://github.com/UMMS-Biocore/dolphinnext	
Software, algorithm	STAR v2.1.6	Dobin et al., 2013	https://github.com/alexdobin/STAR	
Software, algorithm	RSEM v1.3.1	Li and Dewey, 2011	http://deweylab.github.io/RSEM/	
Software, algorithm	DESeq2 v1.28.1	Love et al., 2014	https://bioconductor.org/packages/release/bioc/html/DESeq2.html	
Software, algorithm	clusterProfiler v3.16.1	Yu et al., 2012	https://guangchuangyu.github.io/software/clusterProfiler/	

### Human blood collection

As part of a COVID-19 observational study, peripheral blood samples were collected between March 31st and June 3rd of 2020 from 91 adults with SARS-CoV-2 infection, either after admission to Massachusetts General Hospital for the hospitalized cohort, or while at affiliated outpatient clinics for the outpatient cohort. Request for access to coded patient samples was reviewed by the Massachusetts Consortium for Pathogen Readiness (https://masscpr.hms.harvard.edu/) and approved by the University of Massachusetts Medical School IRB (protocol #H00020836). Pediatric participants with COVID-19 or MIS-C were enrolled in the Massachusetts General Hospital Pediatric COVID-19 Biorepository (MGB IRB # 2020P000955). Healthy pediatric controls were enrolled in the Pediatric Biorepository (MGB IRB # 2016P000949). Samples were collected after obtaining consent from the patient if 18 years or older, or from the parent/guardian, plus assent when appropriate. Demographic, laboratory, and clinical outcome data were included with the coded samples. Samples from 86 adult blood donors and 17 pediatric blood donors were included as controls; these were either collected prior to the SARS-CoV-2 outbreak or from healthy individuals screened at a blood bank.

### Isolation of human peripheral blood mononuclear cells (PMBCs)

Human peripheral blood was diluted in an equal volume of RPMI-1640 (Gibco), overlaid on Lymphoprep (STEMCELL Technologies, #07851), and centrifuged at 500 x g at room temperature for 30 min. Mononuclear cells were washed three times with MACS buffer (0.5% BSA and 2 mM EDTA in PBS) and frozen in FBS containing 10% DMSO.

### Flow cytometry

PBMCs were first stained with Live and Dead violet viability kit (Invitrogen, L-34963). To detect surface molecules, cells were stained in MACS buffer with antibodies (Supplementary file 1a) for 30 min at 4°C in the dark. To detect IL-13 or AREG, cells were stimulated with PMA and ionomycin (eBioscience, 00-4970-03) for 3 hr with Brefeldin A and Monensin (eBioscience, 00-4980-03) present during the stimulation. To detect transcription factors or cytokines, cells were fixed and permeabilized using Foxp3 staining buffer kit (eBioscience, 00-5523-00), then intracellular molecules were stained in permeabilization buffer with antibodies. Cells were detected on a BD Celesta flow cytometer using previously established gating strategies (Wang et al., 2020). Cell subsets were identified using FlowJo software (Becton, Dickson and Company). Representative gating strategies are shown in Figure 2—figure supplement 1.

### Bulk RNA-Seq library preparation

The sequencing libraries were prepared using CEL-Seq2 (Hashimshony et al., 2016). RNA from sorted cells was extracted using TRIzol reagent (ThermoFisher, 15596018). Ten ng RNA was used for first strand cDNA synthesis using barcoded primers (the specific primers for each sample were listed in Supplementary file 1b). The second strand was synthesized by NEBNext Second Strand Synthesis Module (NEB, E6111L). The pooled dsDNA was purified with AMPure XP beads (Beckman Coulter, A63880), and subjected to in vitro transcription (IVT) using HiScribe T7 High Yield RNA Synthesis Kit (NEB, E2040S), then treated with ExoSAP-IT (Affymetrix, 78200). IVT RNA was fragmented using RNA fragmentation reagents (Ambion) and underwent another reverse transcription step using random hexamer RT primer-5’-GCC TTG GCA CCC GAG AAT TCC ANN NNN N-3’ to incorporate the second adapter. The final library was amplified with indexed primers: RP1 and RPI1 (Supplementary file 1b), and the bead purified library was quantified with 4,200 TapeStation (Agilent Technologies) and paired end sequenced on Nextseq 500 V2 (Illumina), Read 1: 15 cycles; index 1: 6 cycles; Read 2: 60 cycles.

### Analysis of RNA-Seq data

Pooled reads from PBMC-derived ILCs were separated by CEL-Seq2 barcodes, and demultiplexed reads from RNA-Seq of ILCs from lung (Ardain et al., 2019), spleen, and intestine (Yudanin et al., 2019), were downloaded from GSE131031 and GSE126107. Within the DolphinNext RNA-Seq pipeline (Revision 4) (Yukselen et al., 2020), reads were aligned to the hg19 genome using STAR (version 2.1.6) (Dobin et al., 2013) and counts of reads aligned to RefSeq genes were quantified using RSEM (version 1.3.1) (Li and Dewey, 2011). Normalized transcript abundance in the form of TPMs were used to filter out low abundance transcripts with an average of <3 TPMs across libraries. RSEM-generated expected counts were normalized and differential analysis was performed using DESeq2 (Love et al., 2014) in R, with significant genes defined as a greater than 1.5-fold difference and an adjusted p-value < 0.01. GO Enrichment Analysis was performed in R using the enrichGO function in the clusterProfiler R package (Yu et al., 2012). Data were transformed using vsd within DESeq2 both for the heatmap visualization with ComplexHeatmap (Gu et al., 2016) and for principal component analysis (PCA) with prcomp on the top 250 most variable genes. Normalized counts were generated for plotting using the counts command in DESeq2.

### Statistical analysis and data visualization

Data were prepared for analysis with tidyverse packages (Wickham et al., 2019) and visualized using the ggplot2 (Wickham, 2016), ggpubr (Kassambara, 2020), and ComplexHeatmap (Gu et al., 2016) packages, within the R computer software environment (version 4.0.2) (R Development Core Team, 2020). Group differences were determined with pairwise, two-sided, Wilcoxon rank-sum tests, or Fisher’s exact test, as indicated, with Bonferroni correction for multiple comparisons. Multiple linear regression analyses were performed with dependent and independent variables as indicated in the text, using the lm function in R. Pairwise group comparisons on estimated marginal means generated from multiple linear regression were performed using the emmeans package (Wieduwilt et al., 2020) in R, with multiple comparison correction using the Tukey adjustment. Multiple logistic regressions were performed using the glm function in R. Longitudinal follow-up analyses on pediatric COVID-19 and MIS-C was performed with linear mixed-effect models using lme4 (Bates et al., 2015) in R with the equation: log_2_ (lymphoid cell abundance) ~ Age + Sex + Group + Group:Follow_up + (1|Patient_ID). This model tested the effect of followup on ILC abundance in the pediatric COVID-19 and MIS-C groups while accounting for age, sex, and group. Statistical significance was determined with lmerTest (Kuznetsova et al., 2017) in R, using the Satterthwarte’s degrees of freedom method. p < 0.05 was considered significant. United States SARS-CoV-2 infection and mortality data were downloaded from (CDC Case Surveillance Task Force, 2020) and cases with age group and outcome available were plotted by age group as indicated. Mortality rate was calculated by dividing the number of fatal cases by the total number of cases with known outcome in each age group as indicated.

## Results

### Characteristics of adult blood donors, either hospitalized for COVID-19, treated for COVID-19 as outpatients, or SARS-CoV-2-uninfected controls

**Figure 1.**
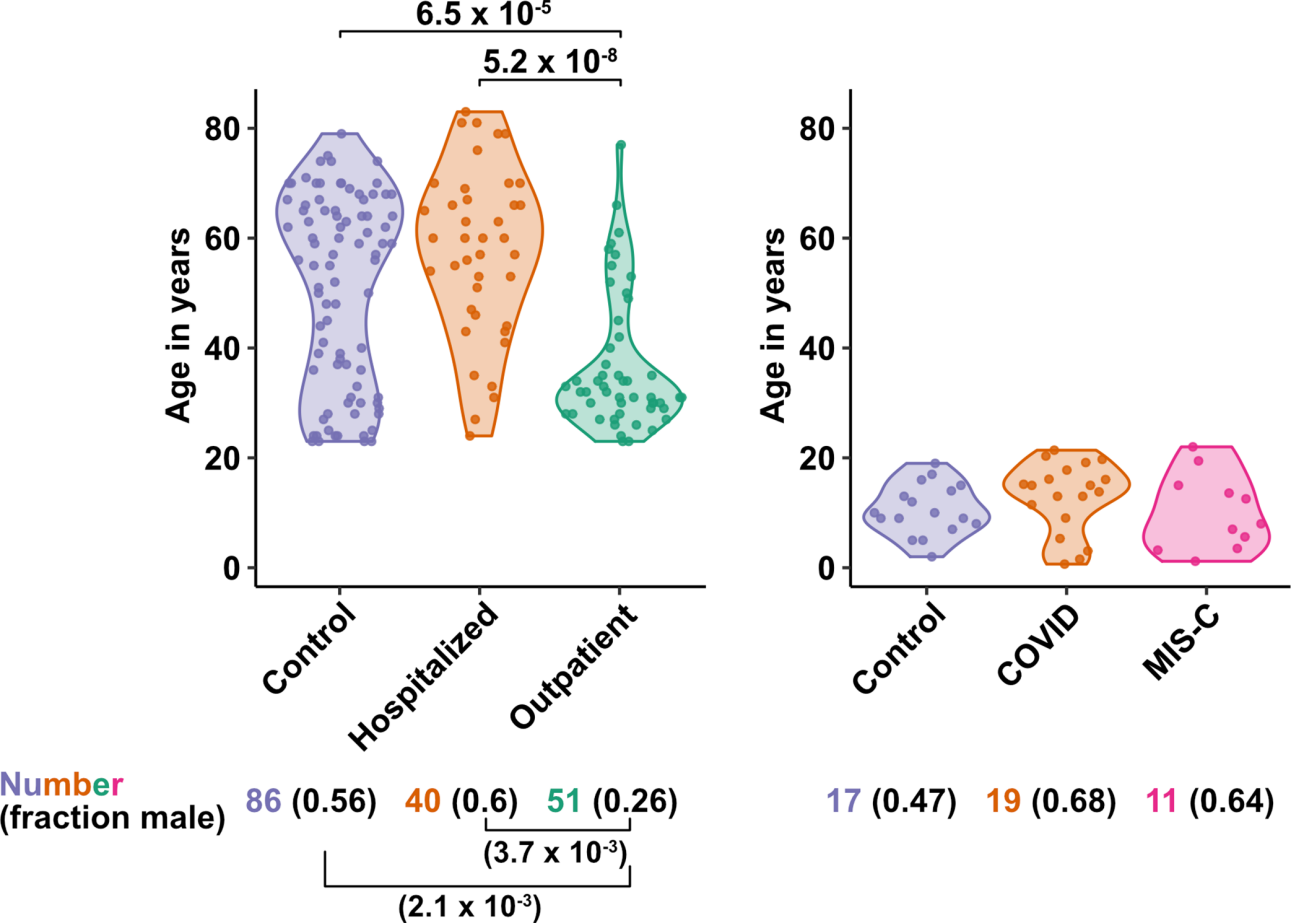
Age and sex of control and SARS-CoV-2-infected blood donors. Age of the subjects is shown, along with the number of subjects and fraction male in each group, for adult (left) and pediatric (right) cohorts, as indicated. The p-values are from pairwise, two-sided, Wilcoxon rank-sum test for ages and Fisher’s exact test for fraction male, with Bonferroni correction for multiple comparisons. Adjusted p-values < 0.05 are shown.

The first group of blood donors in this study included SARS-CoV-2-infected adults hospitalized for severe COVID-19 (N = 40), among whom 33 (82.5%) were admitted to the ICU, 32 (80%) required intubation with mechanical ventilation, and 7 (17.5%) died (Table 1). Aside from intubation, information regarding treatment during hospitalization was not available. This group had a mean age of 57.6 (range 24–83) and 60% were males. The second group consisted of adults infected with SARS-CoV-2 who were treated for COVID-19 as outpatients (N = 51). This group had a mean age of 36.8 years (range 23–77) and was 25.5% male (Table 1). Differences between these two SARS-CoV-2-infected groups, in terms of median age (*P* = 5.2 x 10^–8^), sex ratio (*P* = 3.7 x 10^–3^), and diagnosis of diabetes mellitus (p = 1.05 x 10^–3^), were consistent with established risk factors for severe COVID-19 (Figure 1 and Table 1; Alkhouli et al., 2020; Bunders and Altfeld, 2020; Gupta et al., 2021; Laxminarayan et al., 2020; Mauvais-Jarvis, 2020; O’Driscoll et al., 2021; Peckham et al., 2020; Petrilli et al., 2020; Richardson et al., 2020; Scully et al., 2020). Available information concerning ethnicity and race of the blood donors was insufficient for statistical comparisons among the groups (Supplementary file 2a). Finally, 86 adults who donated blood prior to the SARS-CoV-2 outbreak, or who were screened at a blood donation center, were included as controls for SARS-CoV-2 infection. The age of this group spanned the range of the two groups of SARS-CoV-2-infected people (mean age 50.9; range 23–79), and the percentage of males (55.8%) was similar to that of the group hospitalized for COVID-19 (Table 1 and Figure 1). Complete information regarding ethnicity, race, and comorbidity was not available for control blood donors.

**Table 1.** Demographic and clinical characteristics of adult blood donor groups.

Characteristic	ControlN = **86**	HospitalizedN = **40**	OutpatientN = **51**
Mean age (range) - years	50.9 (23–79)	57.6 (24–83)	36.8 (23–77)
Sex – number (%)			
Male	48 (55.8)	24 (60)	13 (25.5)
Female	38 (44.2)	16 (40)	38 (74.5)
Mean symptom duration at sample collection (range) – days		21.8 (5–66)	26.9 (1–61)
Diabetes mellitus diagnosis – number (%)		11 (27.5)	1 (2)
ICU admission – number (%)		33 (82.5)	
Intubation with mechanical ventilation – number (%)		32 (80)	
Deaths – number (%)		7 (17.5)	
Mean time hospitalized (range) – days		34.2 (4–87)	
Max lab value – mean (range)			
CRP – mg/L		228.6 (6.5–539.5)	
ESR – mm/h		89.0 (15–146)	
D-dimer – (ng/mL)		5700 (351–11923)	

### Characteristics of pediatric blood donors with COVID-19, MIS-C, or SARS-CoV-2-uninfected controls

Children are less likely than adults to have severe disease when infected with SARS-CoV-2 despite having viral loads as high as adults (Bailey et al., 2021; Heald-Sargent et al., 2020; Li et al., 2020; LoTempio et al., 2021; Lu et al., 2020; Poline et al., 2020; Yonker et al., 2020). Rarely, after SARS-CoV-2 clearance from the upper airways, children can develop severe Multisystem Inflammatory Syndrome in Children (MIS-C), a life-threatening condition distinct from COVID-19 that presents with high fevers and multiorgan injury, often including coronary aneurysms, ventricular failure, or myocarditis (Cheung et al., 2020; Feldstein et al., 2021; Feldstein et al., 2020; Licciardi et al., 2020; Riphagen et al., 2020; Verdoni et al., 2020; Whittaker et al., 2020).

The first cohort of pediatric blood donors in this study consisted of patients with COVID-19 who were treated in hospital (N = 11) or as outpatients (N = 8). The second cohort of pediatric blood donors was patients hospitalized for MIS-C (N = 11). Seventeen SARS-CoV-2-uninfected pediatric blood donors constituted a control group. No significant differences in age or percentage of males were detected among the pediatric COVID-19, MIS-C, or pediatric control groups (Supplementary file 2b and Figure 1).

### Blood ILC abundance decreases exponentially across the lifespan and is sexually dimorphic

**Figure 2.**
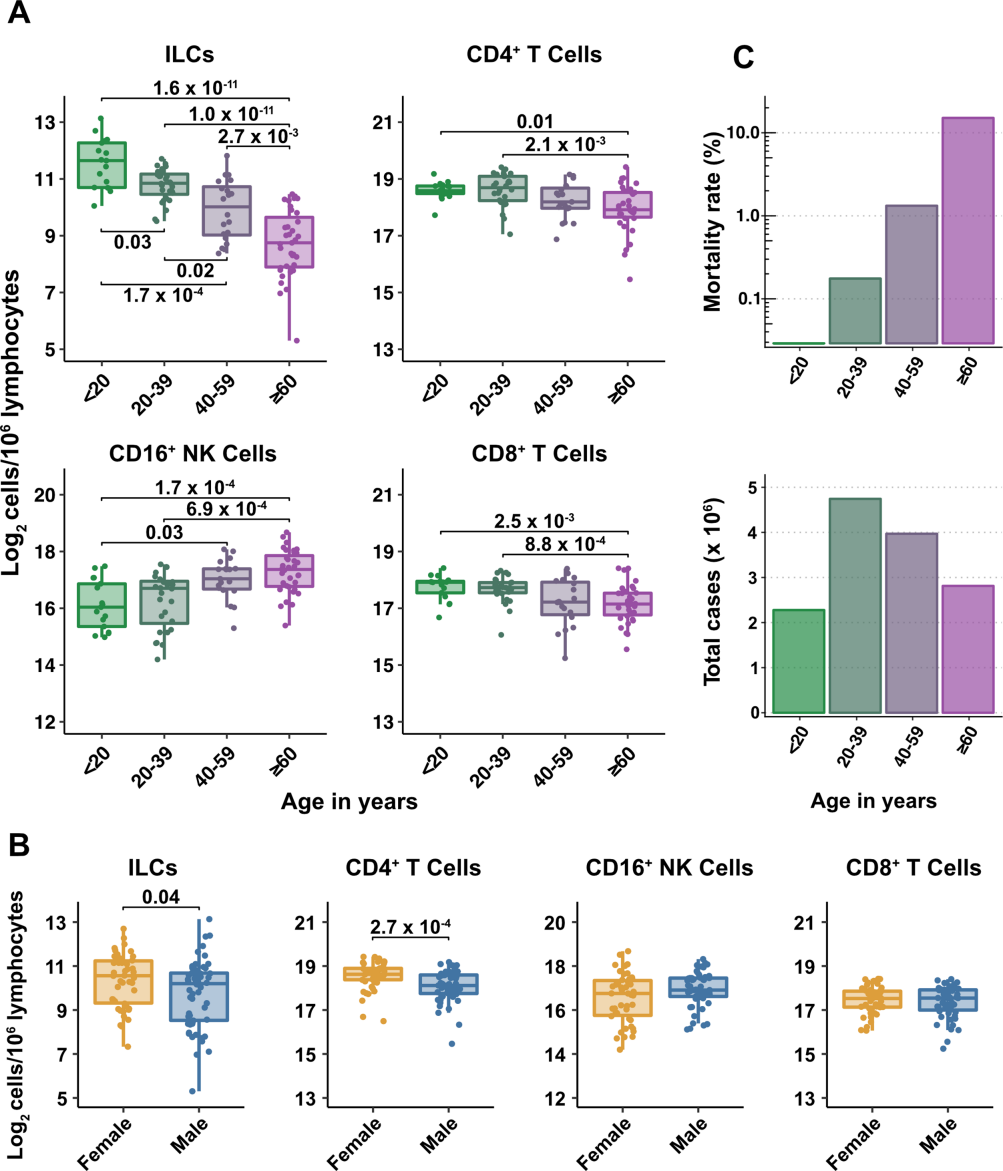
Blood ILC abundance decreases exponentially across the lifespan mirroring the mortality rate from SARS-CoV-2 infection. (**A–B**) Log_2_ abundance per million lymphocytes of the indicated lymphoid cell populations in combined pediatric and adult control data plotted by 20 year bin or by sex, as indicated. Each dot represents an individual blood donor. Boxplots represent the distribution of the data with the center line drawn through the median with the upper and lower bounds of the box at the 75th and 25th percentiles respectively. The upper and lower whiskers extend to the largest or smallest values within 1.5 x the interquartile range (IQR). The p-values are from two-sided, Wilcoxon rank-sum tests with Bonferroni correction for multiple comparisons. Adjusted p-values < 0.05 are shown. (**C**) Case numbers and mortality rate within the indicated age ranges for cases reported in the United States between Jan 1, 2020, and June 6, 2021. Figure 2—source data 1.Combined demographic, clinical, and flow cytometry data for adult COVID-19 and control cohorts.Data included in this file are associated with Figures 1—3; Figure 3—figure supplement 1; Figure 6; Tables 1–4; and Supplementary file 2d,e . File name: Adult_COVIDandControl_data.xlsx.

Lymphoid cell abundance in peripheral blood changes with age and is sexually dimorphic (Márquez et al., 2020; Patin et al., 2018). Previous studies reporting the effect of COVID-19 on the abundance of blood lymphoid cell subsets have not fully accounted for the association of age and sex with COVID-19 severity. To isolate the effect of COVID-19 on cell abundance from effects of age and sex, PBMCs were collected from 103 SARS-CoV-2-negative blood donors distributed from 2 to 79 years of age, with a nearly equal ratio of males to females (Supplementary file 2c). Abundance of lymphoid cell types was plotted by 20-year age groups (Figure 2A), as well as by sex (Figure 2B). Lymphoid cell types assessed here included CD4^+^ T cells, CD8^+^ T cells, ILCs, and FcγRIII (CD16)-positive NK cells. Like CD8^+^ T cells, NK cells kill virus-infected cells using perforin and granzyme (Artis and Spits, 2015; Cherrier et al., 2018). Additionally, by binding virus-specific immunoglobulins that target virus-infected cells for antibody-dependent cellular cytotoxicity, CD16^+^ NK cells link innate and acquired immunity (Anegón et al., 1988).

**Figure 2—figure supplement 1.**
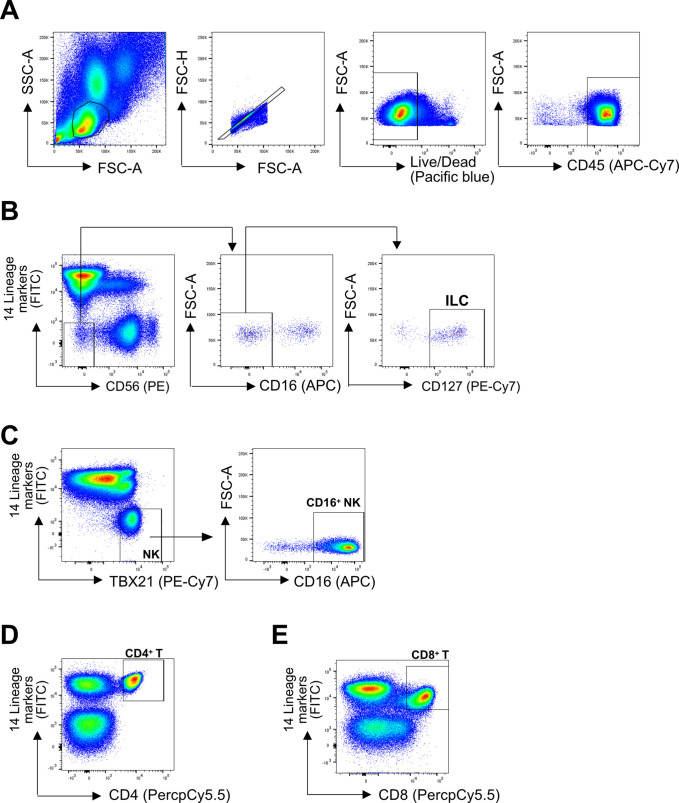
Representative gating strategy. (**A**) All cell subsets were first gated on lymphoid cells, singlets, live/dead, and CD45^+^. Lineage (Lin) markers included antibodies against: CD3, CD4, TCRαβ, TCRγδ, CD19, CD20, CD22, CD34, FcεRIα, CD11c, CD303, CD123, CD1a, and CD14. (**B**) ILCs were identified as Lin^-^CD56^-^CD16^-^CD127^+^ (**C**) CD16^+^ NK cells were identified as Lin^-^TBX21^+^CD16^+^ (**E**) CD4^+^ T cells were identified as Lin^+^CD4^+^ (**D**), and CD8^+^ T cells were identified as Lin^+^CD8^+^.

All cell types examined here were affected by age, but ILCs were the only subset with significant differences among all age groups, falling approximately twofold in median abundance every 20 years, with a greater than sevenfold decrease from the youngest to oldest age groups (p = 1.64 x 10^–11^) (Figure 2A). This magnitude decrease was unique to ILCs and corresponded inversely with the exponential increase in COVID-19 mortality with age (O’Driscoll et al., 2021; Figure 2C). In addition, both ILCs and CD4^+^ T cells were less abundant in males (Figure 2B). These findings highlight the importance of accounting for effects of age and sex when assessing group differences in lymphoid cell abundance, particularly in the context of a disease such as COVID-19 that disproportionately affects older males (O’Driscoll et al., 2021).

### Adults hospitalized with COVID-19 have fewer total lymphocytes even after accounting for effects of age and sex

Severe COVID-19 is associated with lymphopenia (Chen et al., 2020; Huang et al., 2020; Huang and Pranata, 2020; Zhang et al., 2020; Zhao et al., 2020) but it remains unclear if this effect is due to reduction in particular lymphoid cell subpopulations, or whether this effect is explained by the more advanced age and higher proportion of males among people with severe COVID-19. As a first step to assess the specificity of lymphocyte depletion, the effect of COVID-19 on total lymphocyte abundance was addressed with multiple linear regression. After accounting for effects of age and sex, individuals hospitalized with severe COVID-19 had 1.33-fold (95% CI: 1.49–1.19; p = 1.22 x 10^–6^) fewer total lymphocytes among PBMCs than did controls (Supplementary file 2d). Lymphocyte abundance in people infected with SARS-CoV-2 who were treated as outpatients was not different from controls (Supplementary file 2d). In addition, total lymphocytes decreased with age and were less abundant in males (Supplementary file 2d). Subsequent analyses of lymphoid cell subsets took into account the depletion in total lymphocytes associated with COVID-19 by assessing lymphoid subsets as a fraction of total lymphocytes.

### After accounting for age and sex, only innate lymphoid cells are depleted in severe COVID-19

**Figure 3.**
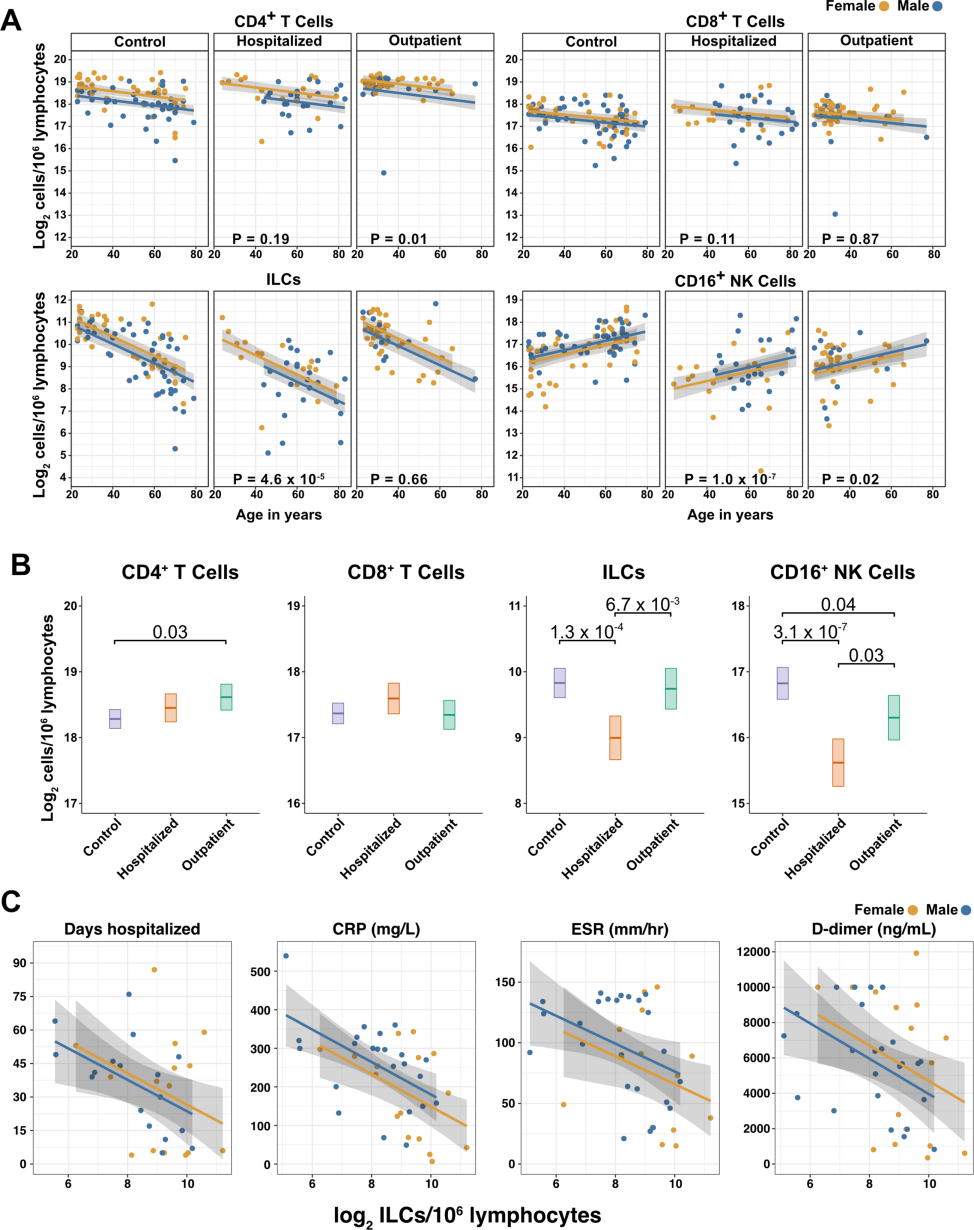
Innate lymphoid cells are depleted in adults hospitalized with COVID-19 and ILC abundance correlates inversely with disease severity. (**A**) Effect of age (X-axis) on log_2_ abundance per million total lymphocytes of the indicated lymphoid cell populations (Y-axis), as determined by the regression analysis in Table 2. Each dot represents an individual blood donor, with yellow for female and blue for male. Shading represents the 95% CI. The p-values are from the regression analysis for comparisons to the control group. (**B**) Log_2_ abundance per million lymphocytes of the indicated lymphoid cell populations, shown as estimated marginal means with 95% CI, generated from the multiple linear regressions in Table 2, and averaged across age and sex. The p-values represent pairwise comparisons on the estimated marginal means, adjusted for multiple comparisons with the Tukey method. Adjusted p-values < 0.05 are shown. (**C**) Association of the indicated clinical parameters with log_2_ abundance of ILCs per million lymphoid cells. Regression lines are from simplified multiple regression models to permit visualization on a two-dimensional plane. Shading represents the 95% CI. Results of the full models accounting for effects of both age and sex, are reported in Table 4 and the text.

To determine whether there were independent associations between lymphoid cell subsets and COVID-19, multiple linear regression was performed on the abundance of lymphoid cell subsets, with age, sex, and group (control, hospitalized, and outpatient) as independent variables. Across all three groups of adult blood donors, CD4^+^ T cells, CD8^+^ T cells, and ILCs decreased with age, while CD16^+^ NK cells increased with age, and both CD4^+^ T cells and ILCs were less abundant in males (Table 2 and Figure 3A).

**Table 2.** Change in cell abundance due to age, sex, and COVID-19 severity fold difference (log_2_) [± 95% CI].

	CD4^+^ T*	ILC*	CD8^+^ T*	CD16^+^ NK*
Age	–0.012***	–0.043***	–0.009*	0.021***
	[–0.018,–0.005]	[–0.053,–0.033]	[–0.016,–0.002]	[0.010, 0.032]
Male	–0.409***	–0.334*	–0.177	0.184
	[–0.618,–0.201]	[–0.659,–0.010]	[–0.406, 0.051]	[–0.169, 0.538]
Hospitalized	0.168	–0.835***	0.227	–1.205***
	[–0.084, 0.421]	[–1.228,–0.441]	[–0.050, 0.503]	[–1.633,–0.778]
Outpatient	0.332*	–0.088	–0.023	–0.522*
	[0.082, 0.581]	[–0.478, 0.302]	[–0.298, 0.253]	[–0.948,–0.095]
R^2^	0.275	0.478	0.070	0.232

*p< 0.05, **p < 0.01, ***p < 0.001.

* per 10^6^ lymphocytes.

**Figure 3—figure supplement 1.**
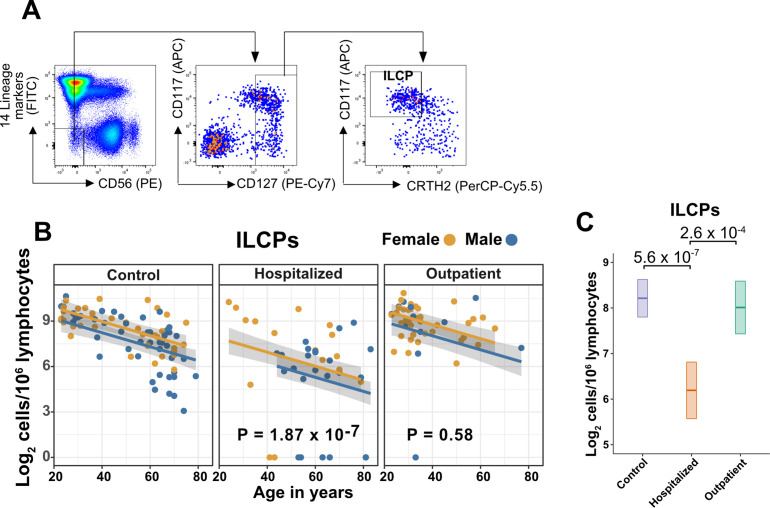
Innate lymphoid cell precursors (ILCPs) decrease with age and are depleted in patients hospitalized with COVID-19. (**A**) Representative gating for ILCP identification in PBMCs. Cells were first gated on lymphoid cells, singlets, live/dead, and CD45^+^. Lineage (Lin) markers included antibodies against: CD3, CD4, TCRαβ, TCRγδ, CD19, CD20, CD22, CD34, FcεRIα, CD11c, CD303, CD123, CD1a, and CD14. (**B**) Effect of age (X-axis) on log_2_ ILCP abundance per million total lymphocytes (Y-axis). Each dot represents an individual blood donor, with yellow for female and blue for male. Shading represents the 95% CI. The p-values are from the regression analysis for comparisons to the control group. (**C**) Lymphoid cell abundance by group, shown as estimated marginal means with 95% CI, generated from the multiple linear regressions in (**B**), and averaged across age and sex. The p-values represent pairwise comparisons on the estimated marginal means, adjusted for multiple comparisons with the Tukey method.

**Figure 3—figure supplement 2.**
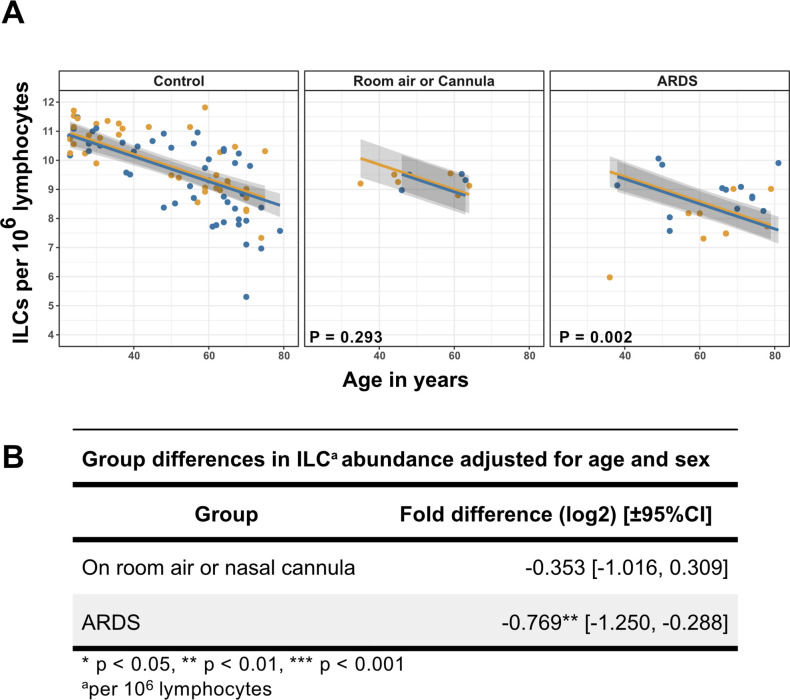
Association of blood ILC depletion with COVID-19 severity in an independent cohort of adults. (**A**) Effect of age (X-axis) on log_2_ ILC abundance per million total lymphocytes (Y-axis) in adult controls from this paper and patients with COVID-19 from Kuri-Cervantes et al., 2020. Patients were stratified into two groups by disease severity. The first group included patients maintained on room air or treated with O2 by nasal cannula. The second group included those with ARDS. Each dot represents an individual blood donor, with yellow for female and blue for male. Shading represents the 95% CI. The p-values are from the regression analysis for comparisons to the control group. (**B**) Table of regression coefficients for log_2_ fold difference in ILC abundance for patients with COVID-19, in comparison to the adult healthy control cohort, adjusted for effects of age and sex.

When effects of age and sex were held constant, adults hospitalized with COVID-19 had 1.78-fold fewer ILCs (95% CI: 2.34–1.36; p = 4.55 x 10^–5^) and 2.31-fold fewer CD16^+^ natural killer (NK) cells (95% CI: 3.1–1.71; p = 1.04 x 10^–7^), as compared to controls (Table 2 and Figure 3B). Similar effects were also seen with ILC precursors (ILCP) (Figure 3—figure supplement 1). Neither CD4^+^ T cells nor CD8^+^ T cells were depleted further than expected for age and sex (Table 2 and Figure 3B). As compared with controls, SARS-CoV-2-infected adults with less severe COVID-19 who were treated as outpatients had no reduction in ILCs, but 1.44-fold fewer CD16^+^ NK cells (95% CI: 1.93–1.07; p = 0.018), and 1.26-fold higher CD4^+^ T cells (95% CI: 1.06–1.5; p = 9.59 x 10^–3^) (Table 2 and Figure 3B). As these analyses were performed on lymphoid cell abundance normalized to total lymphocyte number, it is possible that T cells were not lower in patients hospitalized with COVID-19 because the amount of depletion was not in excess of the change in total lymphocytes. However, the cell-type-specific results remained unchanged even when the analyses were repeated using the less stringent threshold of normalizing to total PBMC number (Supplementary file 2e).

When data from an independent, previously published cohort (Kuri-Cervantes et al., 2020) were analyzed to account for total lymphocyte abundance, age, and sex, people hospitalized with acute respiratory distress syndrome due to COVID-19, had 1.7-fold fewer ILCs (95% CI: 2.38–1.22; p = 0.002) than controls (Figure 3—figure supplement 2). Also consistent with the main adult cohort studied here, ILC abundance was not significantly reduced in the group of patients with less severe disease (Figure 3—figure supplement 2).

### Odds of hospitalization in adults infected with SARS-CoV-2 increases with decreasing number of ILCs

Multiple logistic regression was used next to determine whether differences in abundance of any lymphoid cell subset was associated with odds of hospitalization in people infected with SARS-CoV-2. The adjusted odds ratio was calculated using lymphoid cell subset abundance, age, sex, diagnosis of diabetes mellitus, and duration of symptoms at the time of blood draw, each as independent variables. Abundance of ILCs, but not of CD16^+^ NK cells, CD4^+^ T cells, or CD8^+^ T cells was associated with odds of hospitalization: the odds ratio for hospitalization, adjusted for age, sex, diagnosis of diabetes mellitus, and symptom duration, was 0.454 (95%CI: 0.213–0.808; p = 0.018), an increase of 54.6% for each twofold decrease in ILC abundance (Table 3).

**Table 3.** Odds of hospitalization*.

**Cell count** ^†^	**Odds ratio** ^‡^	**95% Confidence interval**	**p-value**
CD4^+^ T	0.576	0.211–1.28	0.198
ILC	0.454	0.213–0.808	0.018
CD8^+^ T	1.2	0.584–3.06	0.652
CD16^+^ NK	0.841	0.538–1.27	0.412

* Adjusted for age, sex, diagnosis of diabetes mellitus, and symptom duration at time of sample collection.

† per 10^6^ lymphocytes.

‡ per twofold increase in cell population abundance.

### Duration of hospital stay in adults with COVID-19 increases with decreasing ILC abundance

The relationship between lymphoid cell abundance and duration of hospitalization was assessed to determine whether the association between ILC abundance and COVID-19 severity extended to clinical outcomes within the hospitalized adults. This relationship was assessed with multiple linear regression, including age, sex, and cell abundance as independent variables. Holding age and sex constant, abundance of ILCs, but not of CD16^+^ NK cells, CD4^+^ T cells, or CD8^+^ T cells, was associated with length of time in the hospital: each twofold decrease in ILC abundance was associated with a 9.38-day increase in duration of hospital stay (95% CI: 15.76–3.01; p = 0.0054) (Figure 3C and Table 4).

**Table 4.** Association of cell type abundance with time in hospital and laboratory values*.

Cell count^†^	Days hospitalized	CRP (mg/L)^‡^	ESR (mm/h)^‡^	D-dimer (ng/mL)^‡^
**CD4^+^ T**	–10.843	–3.335	–2.674	–1868.847*
	[–22.511, 0.825]	[–56.162, 49.492]	[–23.840, 18.492]	[–3375.630, –362.063]
**ILC**	–9.381**	–46.288***	–11.035*	–1098.515*
	[–15.755,–3.008]	[–71.337, –21.238]	[-21.936,–0.134]	[–1932.842, –264.188]
**CD8^+^ T**	3.366	32.247	15.317	486.192
	[–8.992, 15.724]	[–16.509, 81.003]	[–4.127, 34.761]	[–1049.836, 2022.221]
**CD16^+^ NK**	–4.775	–14.619	–5.159	–404.873
	[–11.251, 1.701]	[–44.011, 14.774]	[–16.809, 6.491]	[–1316.261, 506.516]

* p< 0.05, ** p < 0.01, *** p < 0.001.

* coefficients are for each two-fold increase in cell population abundance, adjusted for age and sex [ ± 95% CI].

† per 10^6^ lymphoid cells.

‡ Maximum lab value recorded during course of hospitalization.

### ILC abundance correlates inversely with markers of inflammation in adults hospitalized with COVID-19

To further characterize the extent to which lymphoid cell abundance predicted COVID-19 severity, multiple regression with age, sex, and cell abundance, as independent variables, was performed on peak blood levels of inflammation markers indicative of COVID-19 severity: C-reactive protein (CRP) and erythrocyte sedimentation rate (ESR), and the fibrin degradation product D-dimer (Gallo Marin et al., 2021; Gupta et al., 2021; Luo et al., 2020; Zhang et al., 2020; Zhou et al., 2020). Holding age and sex constant, each two-fold decrease in ILC, but not in CD16^+^ NK cell, CD4^+^ T cell, or CD8^+^ T cell abundance, was associated with a 46.29 mg/L increase in blood CRP (95% CI: 71.34–21.24; p = 6.25 x 10^–4^) and 11.04 mm/hr increase in ESR (95% CI: 21.94–0.13; p = 0.047) (Figure 3C and Table 4). Abundance of both ILCs and CD4^+^ T cells was associated with blood levels of D-dimer, with each two-fold decrease in cell abundance associated with an increase in D-dimer by 1098.52 ng/mL (95% CI: 1932.84–264.19; p = 0.011) and 1868.85 ng/mL (95% CI: 3375.63–362.06; p = 0.016), respectively (Table 4).

### ILCs are depleted in children and young adults with COVID-19 or MIS-C

**Figure 4.**
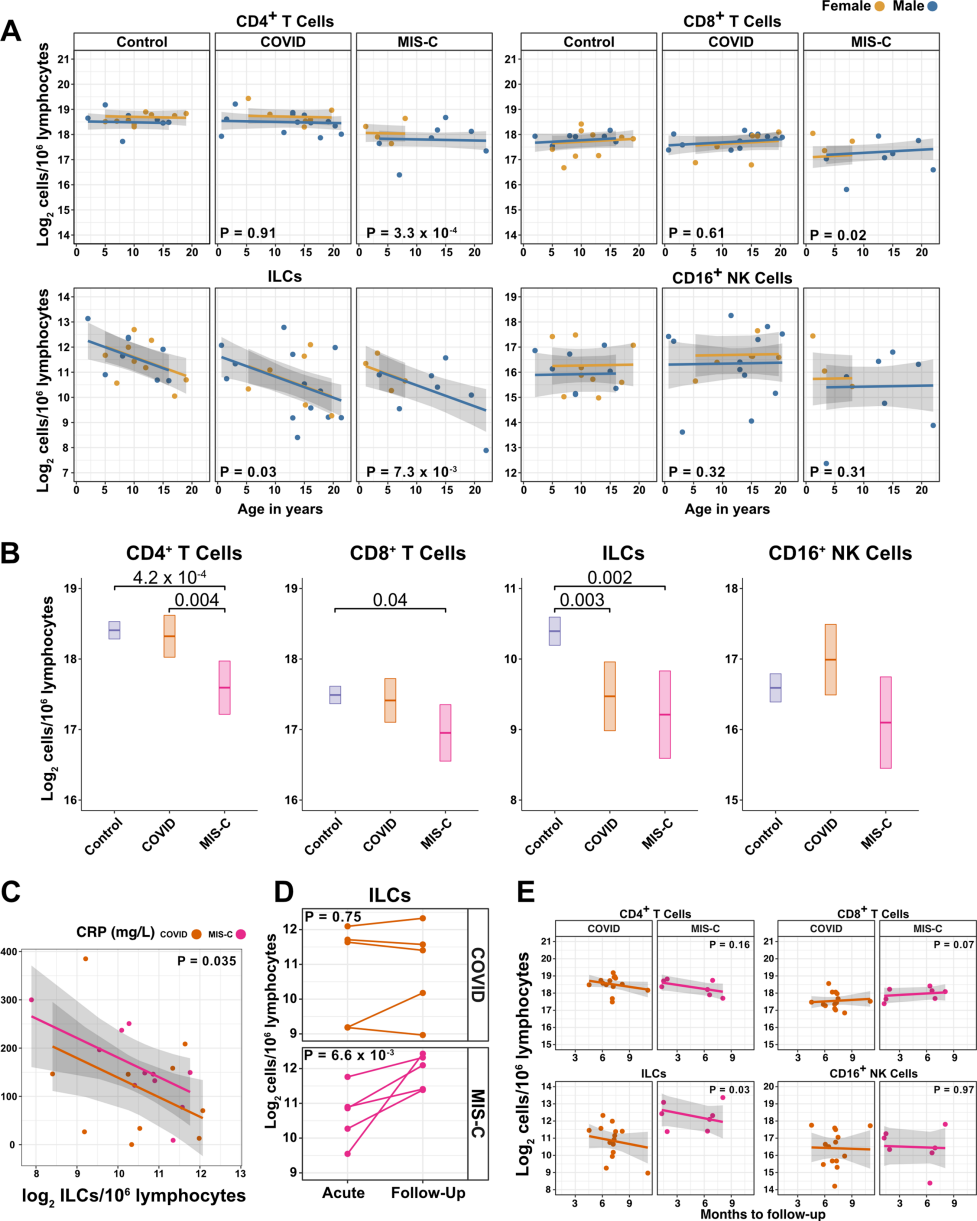
ILCs are depleted in children with COVID-19 or MIS-C. (**A**) Effect of age (X-axis) on log_2_ abundance per million lymphocytes of the indicated lymphoid cell populations (Y-axis), as determined by the regression analysis in Table 5. Each dot represents an individual blood donor, with yellow for female and blue for male. Shading represents the 95% CI. The p-values are from the regression analysis for comparisons to the control group. (**B**) Log_2_ abundance per million lymphocytes of the indicated lymphoid cell populations, shown as estimated marginal means with 95% CI, generated from the multiple linear regressions in Supplementary file 2g that included the combined pediatric and adult control data, and averaged across age and sex. The p-values represent pairwise comparisons on the estimated marginal means, adjusted for multiple comparisons with the Tukey method. Adjusted p-values < 0.05 are shown. (**C**) Association of CRP with log_2_ abundance of ILCs per million lymphocytes. Shading represents the 95% CI. Each dot represents a single blood donor, orange for COVID-19, magenta for MIS-C. The p-value is for the effect of ILC abundance on CRP as determined by linear regression. (**D**) Log_2_ ILC abundance per million lymphocytes in longitudinal pairs of samples collected during acute presentation and during follow-up, from individual children with COVID-19 or MIS-C. Each pair of dots connected by a line represents an individual blood donor. The p-values are for change in ILC abundance at follow-up, as determined with a linear mixed model, adjusting for age, sex, and group, and with patient as a random effect. (**E**) Effect of time to follow-up (X-axis) on log_2_ abundance per million lymphocytes of the indicated lymphoid cell populations (Y-axis). The p-values are for the difference between the COVID-19 and MIS-C follow-up groups, independent of time to follow-up as determined by multiple linear regression. Shading represents the 95% CI. Figure 4—source data 1.Combined demographic, clinical, and flow cytometry data for pediatric COVID-19, MIS-C, and control cohorts.Data included in this file are associated with Figures 1 and 4; Figure 4—figure supplement 1; Figure 4—figure supplement 2; Table 5, and Supplementary file 2f,g. File name: Pediatric_ COVID_MISC_andControl_data.xlsx.

Given the decline in ILC abundance with age (Figures 2A and 3A, and Table 2), and the inverse relationship between ILC abundance and disease severity in adults (Figure 3C, and Tables 3 and 4), it was hypothesized that children as a group have less severe COVID-19 because ILC abundance is higher at younger ages, and that pediatric cases with symptomatic SARS-CoV-2 infection, or with MIS-C, are accompanied by significantly lower numbers of ILCs. To test these hypotheses, the abundance of lymphoid cell subsets in pediatric COVID-19 or MIS-C was compared with that from pediatric controls, using multiple linear regression with age, sex, and group as independent variables. Consistent with the findings in adults, blood ILCs in the pediatric cohort decreased with age (Table 5 and Figure 4A), demonstrating that the decrease in ILC abundance across the lifespan is already evident within the first two decades of life. In contrast, significant change over this age range was not detected in the abundance of CD4^+^ T cells, CD8^+^ T cells, or CD16^+^ NK cells (Table 5 and Figure 4A).

**Figure 4—figure supplement 1.**
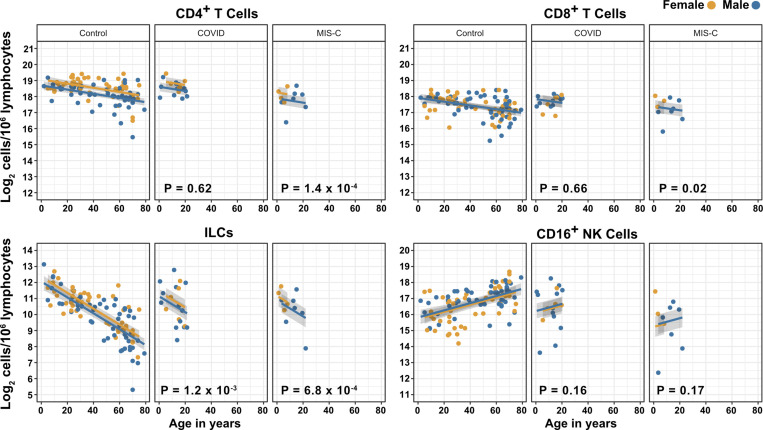
Effects of pediatric COVID-19 and MIS-C on blood lymphoid cell subsets in comparison to full combined adult and pediatric control group. Effect of age (X-axis) on log_2_ abundance per million total lymphocytes of the indicated lymphoid cell populations (Y-axis). Each dot represents an individual blood donor, with yellow for female and blue for male. Shading represents the 95% CI. The p-values are from the regression analysis for comparisons to the control group.

**Figure 4—figure supplement 2.**
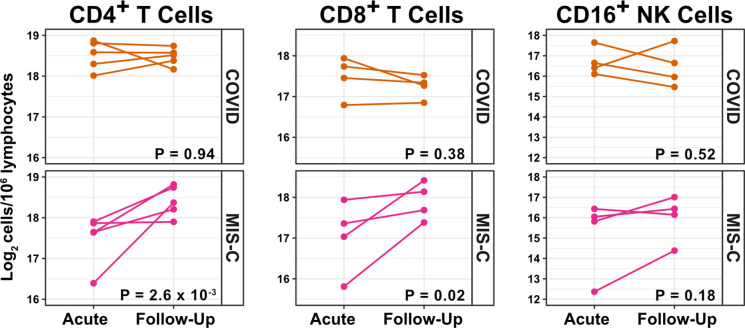
T cells increase during follow-up from MIS-C. Log_2_ ILC abundance per million lymphocytes in longitudinal pairs of samples collected during acute presentation and during follow-up, from individual children with COVID-19 or MIS-C. The p-values are for change in ILC abundance at follow-up, as determined with linear mixed models, adjusting for age, sex, and group, and with patient as a random effect. Differences in sample size among cell types was due to limited sample availability.

**Table 5.** Change in pediatric cohort cell abundance due to age, sex, and group. Fold difference (log_2_) [± 95% CI].

	CD4^+^T*	ILC*	CD8^+^T*	CD16^+^NK*
Age	–0.004	–0.083**	0.012	0.004
	[–0.027, 0.019]	[–0.135,–0.032]	[–0.014, 0.039]	[–0.060, 0.068]
Male	–0.219	–0.027	0.060	–0.343
	[–0.492, 0.054]	[–0.640, 0.586]	[–0.249, 0.370]	[–1.098, 0.413]
COVID	0.018	–0.754*	–0.088	0.416
	[–0.290, 0.327]	[–1.447,–0.061]	[–0.432, 0.257]	[–0.424, 1.257]
MIS-C	–0.678***	–1.098**	–0.503*	–0.498
	[–1.028,–0.328]	[–1.884,–0.313]	[–0.904,–0.101]	[–1.479, 0.483]
R^2^	0.359	0.342	0.169	0.106

* p < 0.05, ** p < 0.01, *** p < 0.001.

* ^a^per 10^6^ lymphocytes.

Among pediatric patients with COVID-19, no difference in abundance of the lymphoid cell subsets was associated with hospitalization (Supplementary file 2f), so all pediatric patients treated for COVID-19 were analyzed as a single group. After accounting for effects of age and sex, pediatric patients with COVID-19 had 1.69-fold fewer ILCs (95% CI: 2.73–1.04; p = 0.034) than controls (Figure 4A and Table 5). Neither CD4^+^ T cells, CD8^+^ T cells, nor CD16^+^ NK cells were depleted in pediatric COVID-19 patients (Figure 4A and Table 5).

As with pediatric COVID-19, ILCs were also lower in MIS-C, with 2.14-fold fewer ILCs (95% CI: 3.69–1.24; p = 0.007) than controls (Figure 4A and Table 5). However, unlike pediatric COVID-19, individuals with MIS-C had reduced numbers of T cells as compared with pediatric controls, with 1.6-fold fewer CD4^+^ T cells (95% CI: 2.04–1.26; p = 3.28 × 10^–4^) and 1.42-fold fewer CD8^+^ T cells (95% CI: 1.87–1.07; p = 0.016) (Figure 4A and Table 5). Depletion of T cells, then, distinguished MIS-C from both pediatric and adult COVID-19. Additionally, consistent with the finding in adults hospitalized with COVID-19 (Figure 3C and Table 4), after accounting for effect of group, each twofold decrease in ILC abundance in pediatric patients hospitalized with COVID-19 or MIS-C was associated with a 40.5 mg/L increase in blood CRP (95% CI: 77.87–3.13; p = 0.035) (Figure 4C), and no such association was detected with CD4^+^ T cells, CD8^+^ T cells, or CD16^+^ NK cells.

The above analysis of lymphoid cell subsets in pediatric COVID-19 and MIS-C was performed in comparison to pediatric controls alone. Results were essentially unchanged when multiple linear regression was repeated with combined pediatric and adult control groups (Figure 4B, Figure 4—figure supplement 1, and Supplementary file 2g).

### Pediatric MIS-C is distinguished from COVID-19 by recovery of ILCs during follow-up

The availability of follow-up samples in this pediatric cohort provided the opportunity to assess the abundance of lymphoid subsets after recovery from illness. To this end, a linear mixed model was fit to determine the change in ILC abundance from acute illness to follow-up in 10 individuals (5 COVID-19 and 5 MIS-C) for whom both acute and follow-up samples were available. After accounting for effects of age, sex, and group, individuals recovering from MIS-C had a 2.39-fold increase in ILC abundance (95% CI: 1.49–3.81; p = 6.6 × 10^–3^) but there was no significant change in ILC abundance for individuals recovering from COVID-19 (Figure 4D). Both CD4^+^ and CD8^+^ T cells, which were depleted in MIS-C but not in COVID-19, also increased during recovery from MIS-C and remained unchanged during recovery from COVID-19 (Figure 4—figure supplement 2).

The relationship between time to follow-up and lymphoid cell abundance was then examined for all available follow-up samples whether or not a paired sample from the acute illness was available (COVID-19, N = 14; MIS-C, N = 7). This analysis found no relationship between time to follow-up and abundance of any lymphoid subset, and that individuals recovering from MIS-C had 2.28-fold more ILCs (95% CI: 1.11–4.69; p = 0.0265) than individuals recovering from COVID-19 (Figure 4E). Of note, ILC abundance rebounded by 2 months of follow-up for the MIS-C patients, whereas ILC abundance still had not rebounded after 9 months of follow-up for the COVID-19 patients. There was no difference between the follow-up groups in CD4^+^ T cells, CD8^+^ T cells, or CD16^+^ NK cells. Interestingly, prior to being hospitalized with MIS-C, only one of these patients had COVID-19 symptoms and, despite low ILC abundance in the COVID-19 follow-up cohort, only 28.6% of this group had been ill enough to require hospitalization (Supplementary file 2b).

Differences between COVID-19 and MIS-C in regard to T cell depletion and ILC recovery during follow-up indicate that the underlying processes causing lower ILC abundance in these two SARS-CoV-2-associated diseases are different.

### Blood ILCs resemble homeostatic ILCs isolated from lung

In response to the above results with blood ILCs, attempts were made to profile ILCs from the lungs of people with fatal COVID-19, but isolation of ILCs from available samples was unsuccessful. Given that ILCs circulate from tissues to the bloodstream via the thoracic duct (Buggert et al., 2020), blood ILC levels might reflect tissue-resident cells and serve as a surrogate for lung ILCs. While ILCs might be decreased in the blood as a result of sequestration within the COVID-19-damaged lung (Ardain et al., 2019), reduced numbers of ILCs within the intestinal lamina propria of people living with HIV-1 is paralleled by reduction in blood ILCs (Wang et al., 2020).

**Figure 5.**
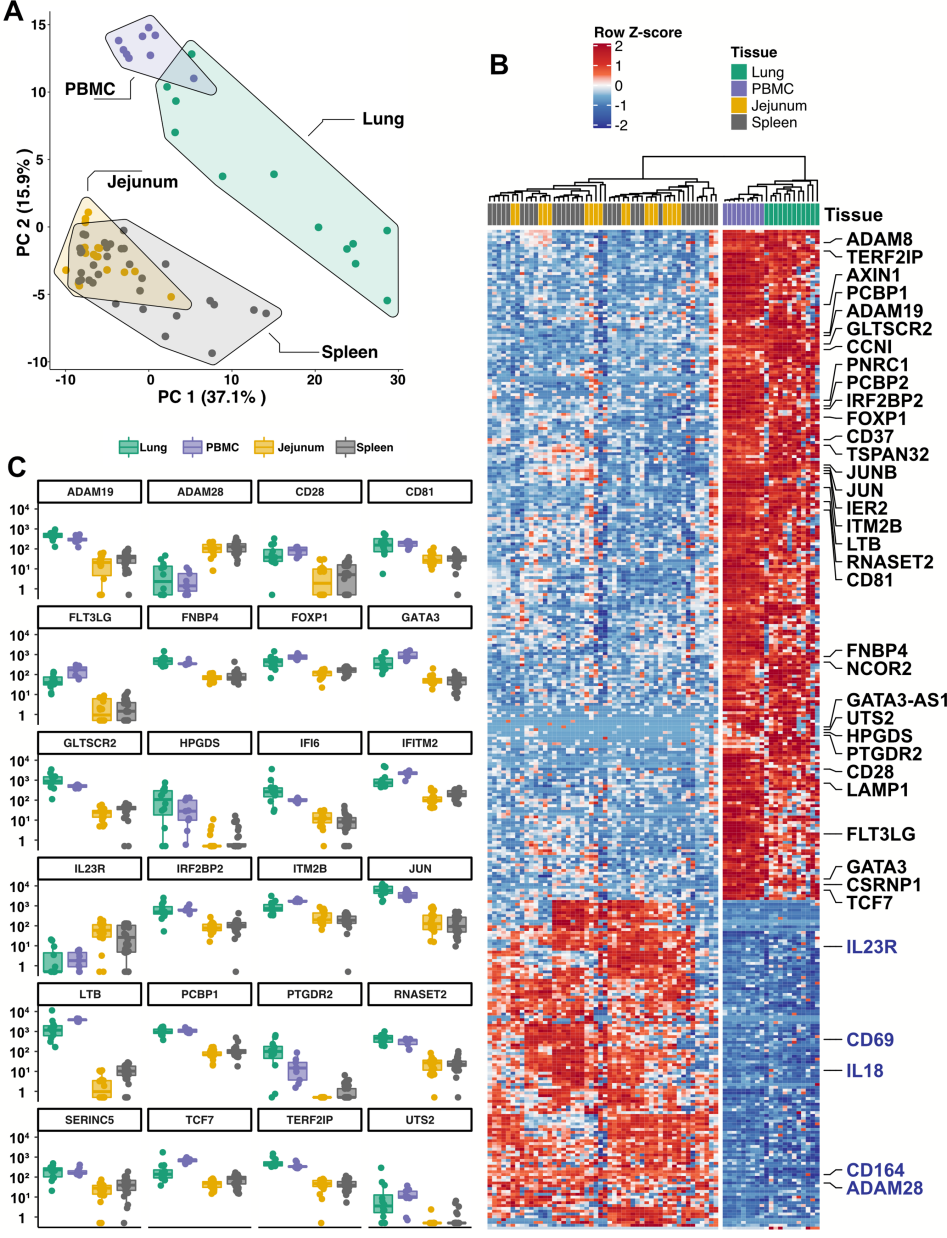
Blood ILCs are transcriptionally similar to lung ILCs.RNA-Seq of ILCs sorted from blood of 9 SARS-CoV-2-uninfected controls in comparison to RNA-Seq data of ILCs sorted from jejunum, lung, and spleen. (**A**) PCA plot of first two principal components calculated from the top 250 most variable genes across all samples. Each dot represents an individual sample with blue for ILCs sorted from blood, green for lung, yellow for jejunum, and grey for spleen. (**B**) Heatmap of 355 genes differentially expressed (fold-change >1.5, padj <0.01 as determined with DESeq2) between either blood or lung ILCs and ILCs from the other tissues. (**C**) Select genes from (**B**) plotted as DESeq2 normalized counts. Each dot represents an individual sample with blue for ILCs sorted from blood, green for lung, yellow for jejunum, and grey for spleen. Boxplots represent the distribution of the data with the center line drawn through the median with the upper and lower bounds of the box at the 75th and 25th percentiles, respectively. The upper and lower whiskers extend to the largest or smallest values within 1.5 x the interquartile range (IQR).

Given the inability to assess lung samples from people with COVID-19, RNA sequencing (RNA-Seq) was performed on blood ILCs from nine healthy controls and these data were compared to previously published RNA-Seq profiles of ILCs sorted from lung, spleen, and intestine (Ardain et al., 2019; Yudanin et al., 2019). Unbiased principal component analysis demonstrated overlap of blood ILCs with ILCs from the lung, with clear separation from ILCs of jejunum or spleen origin (Figure 5A).

Based on expression of characteristic transcription factors and specific inducible cytokines, ILCs are classified into ILC1, ILC2, and ILC3 subsets that are analogous to T_H_1, T_H_2, and T_H_17 cells, respectively (Artis and Spits, 2015; Cherrier et al., 2018; Vivier et al., 2018; Yudanin et al., 2019). A total of 355 genes were consistently differentially expressed (fold-change >1.5, padj <0.01) when either blood or lung ILCs were compared to ILCs from the other tissues (Figure 5B and C). Gene ontology analysis demonstrated enrichment for terms associated with type two immunity (Supplementary file 2h). Genes significantly higher in both blood and lung ILCs included the ILC2-defining genes GATA3 and PTGDR2 (CRTH2), as well as other genes important for ILC development such as TCF7 (Yang et al., 2013; Figure 5B and C).

**Figure 6.**
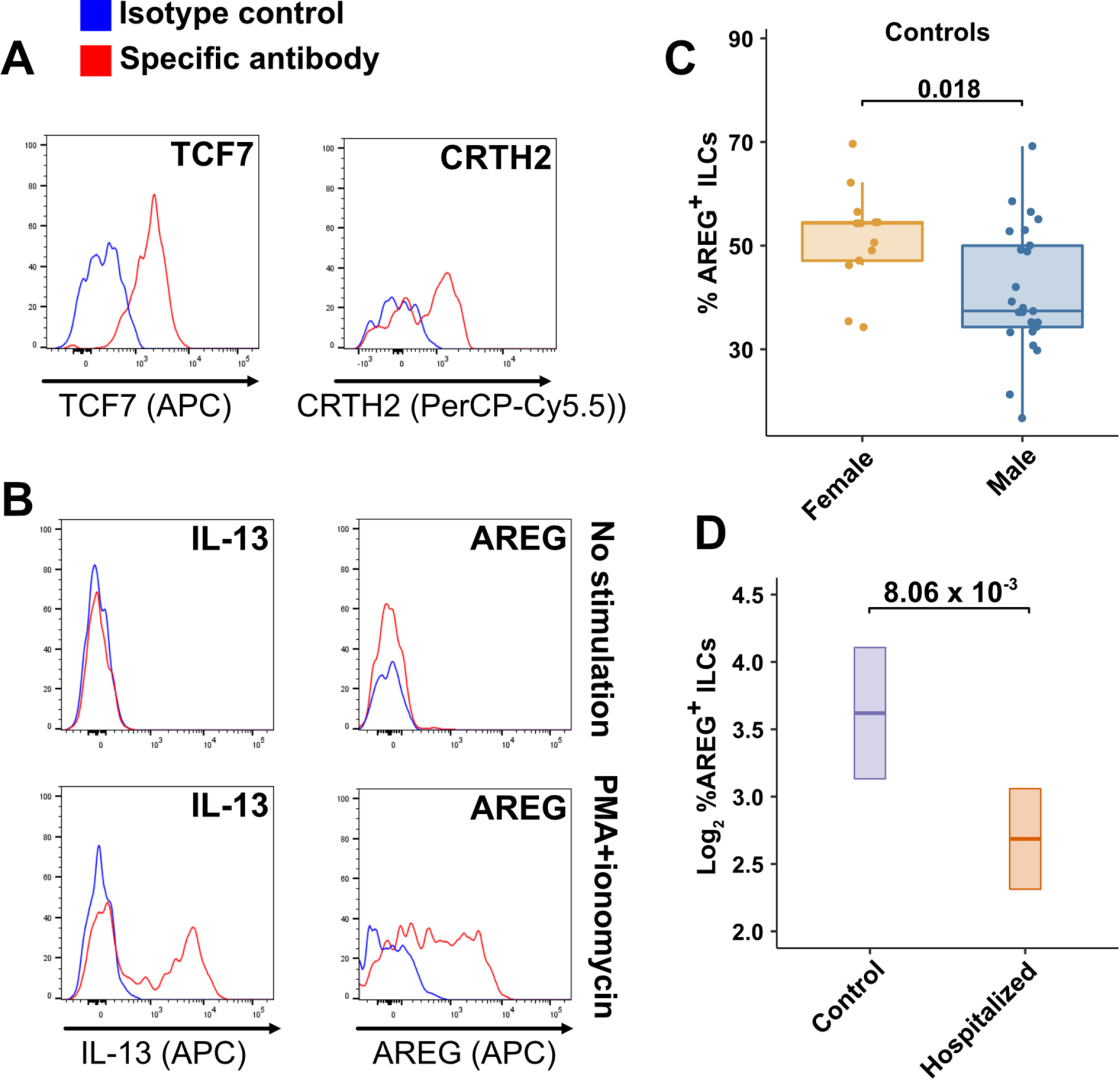
Peripheral blood ILCs exhibit homeostatic ILC2 functions. (**A–B**) Flow cytometry for the indicated proteins. Cells in (**A**) were assayed at steady-state and cells in (**B**) were assayed either at steady-state or after stimulation with PMA and ionomycin, as indicated. Detection of surface proteins was performed on ILCs gated as Lin^-^CD56^-^CD127^+^ and detection of intracellular proteins was performed on ILCs gated as Lin^-^TBX21^-^CD127^+^. (**C**) Percent of AREG^+^ ILCs in blood of control blood donors after stimulation with PMA and ionomycin and gated as Lin^-^TBX21^-^CD127^+^. Each dot represents an individual blood donor (N Female = 13, N Male = 25). Boxplots represent the distribution of the data with the center line drawn through the median with the upper and lower bounds of the box at the 75th and 25th percentiles respectively. The upper and lower whiskers extend to the largest or smallest values within 1.5 x the interquartile range (IQR). The p-value is from a two-sided, Wilcoxon rank-sum test. (**D**) Log_2_ percent AREG^+^ ILCs in blood of controls or people hospitalized with COVID-19 after stimulation with PMA and ionomycin and gated as Lin^-^TBX21^-^. Data shown as estimated marginal means with 95% CI, generated from the multiple linear regression reported in the text and averaged across age and sex. The p-value is from the regression analysis. Figure 6—source data 1.Amphiregulin (AREG) flow cytometry data presented in Figure 6 and Figure 6—figure supplement 1.File name: AREG_in_ILCs.xlsx.

TCF7- and CRTH2-encoded proteins were detected in blood ILCs by flow cytometry, confirming the RNA signature of ILC2s (Figure 6A). To assess the function of blood ILCs, PBMCs were stimulated with PMA and ionomycin, and assayed by flow cytometry for production of IL-13 after intracellular cytokine staining and gating on ILCs. IL-13 was detected in the stimulated ILC population (Figure 6B), demonstrating that the majority of blood ILCs function as ILC2s. Additionally, the blood ILCs produced amphiregulin (Figure 6B), a protein implicated in the promotion of disease tolerance by ILCs in animal models (Branzk et al., 2018; Diefenbach et al., 2020; Jamieson et al., 2013; McCarville and Ayres, 2018; Monticelli et al., 2015; Monticelli et al., 2011).

**Figure 6—figure supplement 1.**
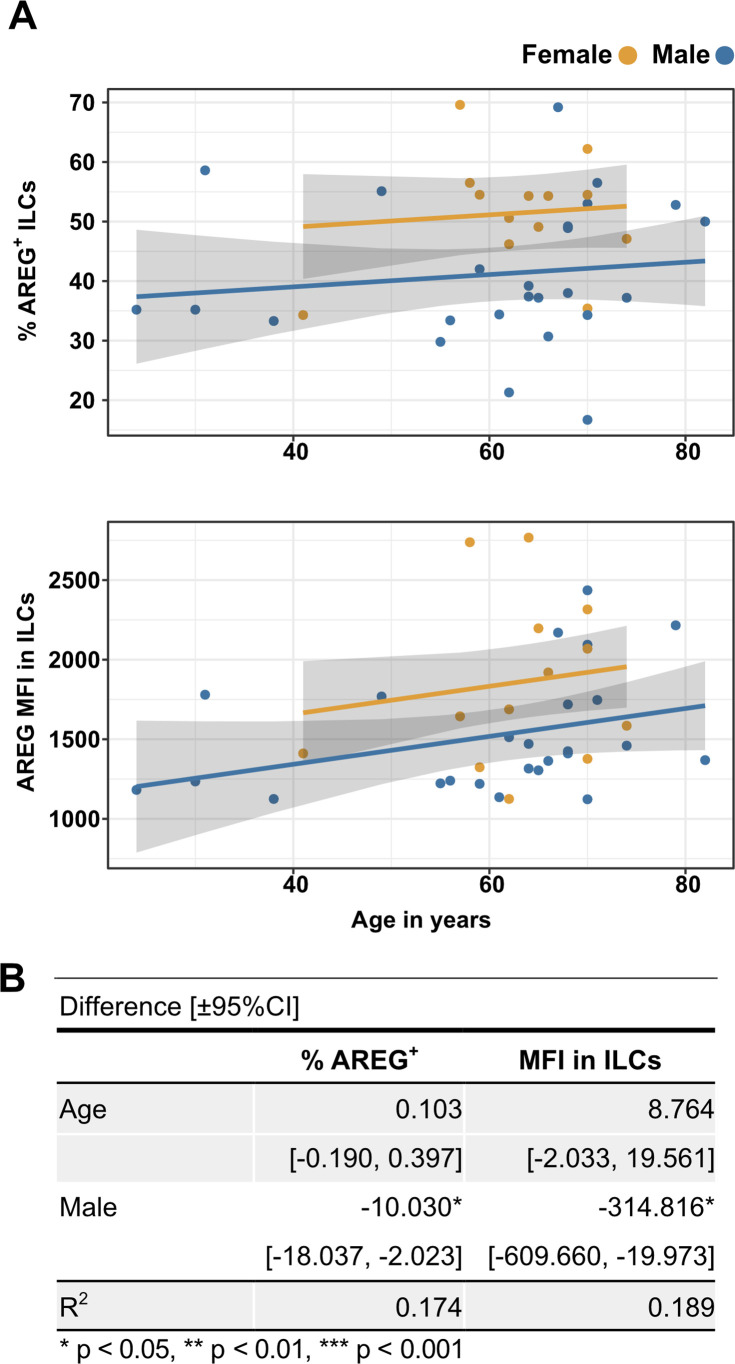
Males have lower percent AREG^+^ ILCs, and lower AREG MFI in ILCs, than do females, and there is no effect of age on these parameters. (**A**) Effect of age (X-axis) on % AREG^+^ ILCs or MFI in ILCs (Y-axis as indicated). Each dot represents an individual blood donor, with yellow for female and blue for male. Shading represents the 95% CI. (**B**) Table of results for regression analyses plotted in (**A**).

### Effect of sex and COVID-19 on fraction of blood ILCs that produce amphiregulin

Given the role that AREG-producing ILCs play in maintaining disease tolerance in animal models (Branzk et al., 2018; Diefenbach et al., 2020; McCarville and Ayres, 2018; Monticelli et al., 2015; Monticelli et al., 2011), sex differences in the functional capability of these ILCs could contribute to the greater risk for severe COVID-19 in males (O’Driscoll et al., 2021). To address this hypothesis, ILCs isolated from the peripheral blood of controls were stimulated with PMA and ionomycin, and assayed by flow cytometry for AREG production. Consistent with the apparently lower disease tolerance in males, males had a lower median fraction of AREG^+^ ILCs than did females (p = 0.018) (Figure 6C). This difference was also reflected in a significantly lower AREG Mean Fluorescent Intensity (MFI) in males, and neither fraction of AREG^+^ ILCs nor AREG MFI was affected by age (Figure 6—figure supplement 1). Multiple linear regression was performed, with age, sex, and group as independent variables, to determine whether hospitalization with COVID-19 was associated with differences in the percentage of AREG^+^ ILCs. This analysis showed that, after accounting for effects of age and sex, patients hospitalized with COVID-19 had a 1.91-fold lower percentage of AREG^+^ ILCs (95% CI: 1.19–3.06; p = 8.06 x 10^–3^) than controls (Figure 6D).

## Discussion

The outcome of SARS-CoV-2 infection ranges from entirely asymptomatic to lethal COVID-19 (Cevik et al., 2021; He et al., 2020; Jones et al., 2021; Lee et al., 2020; Lennon et al., 2020; Ra et al., 2021; Richardson et al., 2020; Yang et al., 2021). Yet, viral load does not reliably discriminate asymptomatic from symptomatic or hospitalized populations (Cevik et al., 2021; Jones et al., 2021; Lee et al., 2020; Lennon et al., 2020; Ra et al., 2021; Yang et al., 2021). In contrast, demographic factors, including increasing age and male sex, predict worse outcome of SARS-CoV-2 infection (Alkhouli et al., 2020; Bunders and Altfeld, 2020; Gupta et al., 2021; Laxminarayan et al., 2020; Mauvais-Jarvis, 2020; O’Driscoll et al., 2021; Peckham et al., 2020; Richardson et al., 2020; Scully et al., 2020). These demographic risk factors could be due to sexual dimorphism and changes with aging in composition and function of the human immune system (Darboe et al., 2020; Klein and Flanagan, 2016; Márquez et al., 2020; Patin et al., 2018; Solana et al., 2012). Therefore it is necessary to account for effects of age and sex to determine if there are additional, independent, effects of SARS-CoV-2-associated disease.

This study collected and analyzed 245 blood samples from 177 adult and 58 pediatric patients and controls, spanning the ages of 0.7–83 years, with approximately equal numbers of males and females. It was therefore possible to characterize the independent effects of age, sex, COVID-19, and MIS-C on blood lymphoid cell populations. After accounting for effects of age and sex, ILCs, but not CD4^+^ or CD8^+^ T cells, were lower in individuals hospitalized with COVID-19 when compared with controls (Table 2 and Figure 3A, B). Lower numbers of ILCs were also observed in children with COVID-19 (Table 5 and Figure 4A, B), as well as in an independent cohort of adult patients (Figure 3—figure supplement 2). Among adults infected with SARS-CoV-2, lower abundance of ILCs, but not of the other lymphoid cell subsets, was associated with increased odds of hospitalization, longer duration of hospitalization, and higher blood level of factors associated with systemic inflammation, including CRP (Tables 3 and 4, and Figure 3C). This inverse relationship between ILC abundance and CRP was also evident in children with COVID-19 or MIS-C (Figure 4C).

The identification of reduced ILC numbers as uniquely related to COVID-19 severity is important as these cells mediate disease tolerance in animal models (Artis and Spits, 2015; Branzk et al., 2018; Califano et al., 2018; Diefenbach et al., 2020; McCarville and Ayres, 2018; Monticelli et al., 2015; Monticelli et al., 2011). The results here therefore indicate that loss of ILCs from blood correlates with loss of ILC-associated homeostatic functions, thereby allowing more severe COVID-19. Although this study examined circulating blood lymphoid cells, and does not provide direct information about processes occurring within tissues, transcriptional and functional characterization of blood ILCs demonstrated that these cells are similar to ILCs isolated from lung tissue (Figure 5). Human ILCs circulate in lymphatic fluid draining from the tissues to the blood via the thoracic duct (Buggert et al., 2020), raising the possibility that some ILCs in the blood originate from, or traffic to, lung tissue. Further characterization of these blood ILCs showed that they are functional ILC2s capable of producing the protein AREG (Figure 6A and B). Given the tissue homeostatic role AREG plays in animal models of disease tolerance (Branzk et al., 2018; Diefenbach et al., 2020; Jamieson et al., 2013; McCarville and Ayres, 2018; Monticelli et al., 2015; Monticelli et al., 2011), the discovery here that males have a smaller fraction than females of blood ILCs capable of producing AREG (Figure 6C) could explain why males are at greater risk of death from SARS-CoV-2 infection (O’Driscoll et al., 2021). This sexual dimorphism in ILC function would be amplified further by the lower overall abundance of ILCs in males (Figure 2B and Table 2).

Although the inverse relationship between the number of blood ILCs and severity of COVID-19 suggests that loss of ILC homeostatic function results in breakdown of disease tolerance (Arpaia et al., 2015; Artis and Spits, 2015; Branzk et al., 2018; Diefenbach et al., 2020; McCarville and Ayres, 2018; Monticelli et al., 2015; Monticelli et al., 2011), this observational study cannot determine whether ILC depletion preceded SARS-CoV-2 infection or whether ILC numbers are depleted as a consequence of SARS-CoV-2 infection. However, several observations support the hypothesis that individuals with lower ILC numbers at the time of SARS-CoV-2 infection are at greater risk of developing severe disease. ILC numbers in uninfected controls decrease exponentially with age; this decrease is much larger than that seen with other lymphoid cell types (Figure 2A), and much more closely mirrors the exponential increase in COVID-19 mortality with age (O’Driscoll et al., 2021; Figure 2C). In addition, the greater risk of COVID-19 mortality in males (O’Driscoll et al., 2021) correlates with lower abundance of blood ILCs (Figure 2B and Table 2) and smaller fraction of ILCs capable of producing AREG (Figure 6C). Finally, patients hospitalized with COVID-19 have a smaller fraction of AREG^+^ ILCs than controls (Figure 6D) and conditions independently associated with lower ILC abundance, such as HIV-1 infection (Kløverpris et al., 2016; Wang et al., 2020) and obesity (Brestoff et al., 2015; Yudanin et al., 2019), increase the risk for worse outcomes from SARS-CoV-2 infection (Biccard et al., 2021; Kompaniyets et al., 2021; Tesoriero et al., 2021).

In contrast to individuals with COVID-19, children with MIS-C had lower numbers of T cells as well as ILCs (Table 5 and Figure 4A and B), and longitudinal follow-up samples for pediatric COVID-19 and MIS-C patients showed persistence of low ILC numbers after COVID-19, but normalization of all depleted cell types after recovery from MIS-C (Figure 4D and E and Figure 4—figure supplement 2). These differences imply that the reversible lymphopenia in MIS-C is due to different underlying processes than the more specific and persistent lower ILC abundance seen in individuals with COVID-19. This difference is made more interesting by the fact that none of the children with MIS-C had required hospitalization for COVID-19 and only one experienced any COVID-19 symptoms. The other children with MIS-C were therefore unaware that they had been infected. It is possible that children with pre-existing lower ILC numbers are at risk of developing COVID-19 if infected with SARS-CoV-2, while other factors such as prolonged exposure to SARS-CoV-2 antigens in the gastrointestinal tract (Yonker et al., 2021), or rare inborn errors of immunity (Sancho-Shimizu et al., 2021), promote inflammatory processes in MIS-C that drive nonspecific lymphoid cell depletion, which ultimately normalizes after recovery.

Although ILC depletion and recovery has been reported in rheumatoid arthritis (Rauber et al., 2017), inflammation-driven ILC-depletion is not necessarily reversible, as ILCs appear permanently depleted after HIV-1 infection, possibly by the high levels of common γ-chain cytokines that are present during acute infection (Wang et al., 2020). Better understanding of the processes that drive down ILC abundance in populations susceptible to COVID-19 could potentially allow for development of interventions that increase ILC abundance and restore homeostatic disease tolerance mechanisms.

In conclusion, considering the established functions of ILCs (Artis and Spits, 2015; Branzk et al., 2018; Klose and Artis, 2016; Monticelli et al., 2015; Monticelli et al., 2011), and the host homeostatic responses necessary to survive pathogenic infection (López-Otín and Kroemer, 2021; McCarville and Ayres, 2018; Medzhitov et al., 2012; Schneider and Ayres, 2008), the findings reported here support the hypothesis that loss of disease tolerance mechanisms attributable to ILCs increase the risk of morbidity and mortality with SARS-CoV-2 infection. The findings of this observational study warrant establishment of prospective cohorts to determine whether abundance of ILCs or of other lymphoid cell subsets associated with disease tolerance (Arpaia et al., 2015; Artis and Spits, 2015; Branzk et al., 2018; Diefenbach et al., 2020; McCarville and Ayres, 2018; Monticelli et al., 2015; Monticelli et al., 2011), predict clinical outcome for infection with SARS-CoV-2 or other lethal pathogens. Understanding the mechanisms that allow an individual to tolerate high-level viral replication without experiencing symptoms, and how these mechanisms can fail and thereby allow for progression to severe disease, will provide the foundation for development of therapeutic interventions that maintain health and improve survival of pathogenic viral infection (Ayres, 2020b).

## Data Availability

The data that support the findings of this study are available within the manuscript and in its supplementary information data files. Bulk RNA-Seq datasets generated here can be found at: NCBI Gene Expression Omnibus (GEO): GSE168212. Bulk RNA-Seq data generated by previously published studies are available from NCBI GEO: GSE131031 and GSE126107. This study did not generate unique code. The following dataset was generated: WangY LifshitzLM LubanJ 2021Systematic analysis of innate lymphoid cells and natural killer cells in context of HIV-1 infectionNCBI Gene Expression OmnibusGSE168212 The following previously published datasets were used: LeslieA KhaderS 2019Group 3 innate lymphoid cells mediate early protective immunity against Mycobacterium tuberculosisNCBI Gene Expression OmnibusGSE131031 YudaninN LatorreI CovingtonC 2019Spatial and Temporal Mapping of Human Innate Lymphoid Cells Reveals Elements of Tissue SpecificityNCBI Gene Expression OmnibusGSE12610710.1016/j.immuni.2019.01.012PMC659437430770247

## Additional files

Supplementary file 1. Antibodies and sequencing primers used here. (**a**) Antibodies Used in Flow Cytometry. (b) Primers for bulk RNA-Seq.

Supplementary file 2. Supplementary Tables. (**a**) Race and Ethnicity of Adult Cohorts (b) Demographic and Clinical Characteristics of Pediatric Blood Donor Groups (c) Comparison of sex ratios between control age groups (d) Change in Lymphocyte Abundance Per 10^6^ PBMCs Due to Age, Sex, and COVID-19 Severity in Adult cohorts (e) Change in Lymphoid Cell Abundance Per 10^6^ PBMCs Due to Age, Sex, and COVID-19 Severity (f) Odds of Hospitalization in Pediatric Cohort (g) Change in Pediatric Cohort Lymphoid Cell Abundance Per 10^6^ lymphocytes due to Group; Adjusted for Effects of Age and Sex with Combined Pediatric and Adult Control Data (h) Gene ontology analysis results.

Transparent reporting form
