# Supplementary material for: Innate lymphoid cells and COVID-19 severity in SARS-CoV-2 infection
Source: eLife. 2022 Mar 11;11:e74681. doi: 10.7554/eLife.74681 (PMC9038195; doi:10.7554/eLife.74681)
Supplement: Peer review history

# Editor report: Editor's evaluation

**DOI:** 10.7554/eLife.74681.sa0

**Authors:** Evangelos J Giamarellos-Bourboulis

**Affiliations:** https://ror.org/04gnjpq42 National and Kapodistrian University of Athens, Medical School Greece

The manuscript shows striking data that circulating innate lymphoid cells (ILCs) decline over human age, more in males than in females, and that a further decline in their prominence is associated with severe COVID-19 in both elderly and paediatric situations. While the findings remain correlative as yet, they shed further light on the complex immune dysregulation of COVID-19, and underline the potential importance of innate lymphoid cells.

# Decision letter

**DOI:** 10.7554/eLife.74681.sa1

**Affiliations:** https://ror.org/04gnjpq42 National and Kapodistrian University of Athens, Medical School Greece; https://ror.org/04gnjpq42 National and Kapodistrian University of Athens, Medical School Greece; https://ror.org/04gnjpq42 National and Kapodistrian University of Athens, Medical School Greece

Our editorial process produces two outputs: i) public reviews designed to be posted alongside the preprint for the benefit of readers; ii) feedback on the manuscript for the authors, including requests for revisions, shown below. We also include an acceptance summary that explains what the editors found interesting or important about the work.

**Decision letter after peer review:**

Thank you for submitting your article "Innate lymphoid cells and disease tolerance in SARS-CoV-2 infection" for consideration by *eLife*. Your article has been reviewed by 3 peer reviewers, including Evangelos J Giamarellos-Bourboulis as Reviewing Editor and Reviewer #1, and the evaluation has been overseen by Satyajit Rath as the Senior Editor. The following individual involved in review of your submission has agreed to reveal their identity: Evdoxia Kyriazopoulou (Reviewer #2).

The reviewers have discussed their reviews with one another, and the Reviewing Editor has drafted this to help you prepare a revised submission.

Essential revisions:

– The Introduction is too long and it is based on references of the past before the arrival of COVID-19.

– One main concern is that the authors suggest that depletion of innate lymphoid cells (ILC) is the main driver of the lymphopenia of severe COVID-19. To prove this the authors need to provide the absolute counts of all subpopulations and to provide serial follow-up data. They also need to explain how hypoglobulinemia of patients is explained if depletion involves only ILC. There are at least two publications (PMID 32320677 and 34678611) suggesting defect in antigen presentation and they are not even referenced in this submission.

– The authors need to provide data on the cytokine production capacity of ILC.

– The authors should clarify when exactly blood sampling took place (at admission? during hospitalization? Before any treatment start?).

– According to Table 1 median duration of symptoms is about 20 days which is quiet a long period; normally patients are admitted after the first week. If sampling is not done at admission, administered therapy especially corticosteroids may influence flow cytometry results. If therapy was administered this has to be part of the regression results in Table 3.

– Another concern is the absence in presentation of comorbidities as they can perhaps also influence results. If these data are unavailable, this is for sure a limitation and should be discussed.

– In SARS-CoV2 infected adults, as reported widely, infections were highest in young adults, and mortality was highest in the elderly and increased approximately 1 log for each 20 year age group. Linear regression revealed that age, sex, and ILC and NK cell frequency were inversely correlated with hospitalization, and the association between ILC and NK frequency was retained when age and sex were adjusted for. Subsequent multiple logistic regression analysis showed that adjusting for age, sex, and symptom duration, only ILC frequency was significantly associated with an increased risk of hospitalization, the duration of hospital stay, and an increase in CRP. The same association between ILC frequency, but not other lymphocytes, and COVID was observed in an additional paediatric cohort. However, in rare Paediatric cases who developed MIS-C, both ILCs and T cells were significantly reduced. To link blood ILCs to what is happening on the lung, bioinformatic analysis of sequence data generated from sorted blood ILCS in compared to that of published gut and lung ILC datasets. Although, the authors argue this shows blood ILCs are transcriptionally more closely aligned to lung ILCs, the validity of this analysis is hard to judge without more detail and, as the data does not come from COVID subjects, its value in supporting the manuscript is unclear. Finally, ILCs from uninfected males produced less amphiregulin, important in lung homeostasis and repair.

– The loss of ILCs from circulation has been shown in several diseases, including HIV, TB, and COVID, as has the association between circulating ILCs and age. The strength of this manuscript is in using multiple regression to show that the association between ILC loss and disease severity is retained when age is controlled for, as age is such an important factor in COVID. However, the overall conclusion that elevated circulating ILCs support disease tolerance, while interesting, is not directly supported by any data. For example, a reduction in blood ILCs may indicate the recruitment of these cells to the lung, as has been shown for TB (Ardain et al., Nature 2019). In which case, an increased frequency of blood ILCs may not be protective per se, but just reflective of less lung involvement. Therefore, I suggest the title and abstract be altered to reflect the data presented more directly.

– The study consistently refers to cell abundance but is actually reporting the frequency of cells. It is an important distinction in this context, as the total lymphocyte count declines with age. Therefore, if there are differences in the rates of decline, it can create the impression of an increase in specific subsets which are actually declining but just at a slower than the median (as may be happening with NK cells for example). To avoid any potential confusion, I suggest the authors present and discuss the data as frequencies of total lymphocytes (or PBMC as appropriate).

– For the lymphocyte specific analysis, what is lacking is reporting of the other lymphocyte subsets, such as B-cells. It may not be possible to represent these as individual populations, depending on the markers used, but they could be presented as the remainder of the whole. In this way, the reader can get a sense of what is happening in the lymphocyte compartment as a whole. This is important given that absolute values are not available.

-I am a bit concerned that the synchronization of the paeds sample is too big an issue in this small sample size to give much confidence in the effects observed – the MISC subjects are measured at about 2months and 6-7 months, and the COVID at 6-8 months and one at 11 months. Given this, how much weight do the authors think this analysis carries?

– The authors compare the RNA read-out of the peripheral ILCs and they use previous arrays from lung ILCs to suggest that they are coming from the lung. This is far too arbitrary since this is a different cohort. Ardain et al., sorted ILC2s and ILC3s from the lung whilst Yudanin sorted ILC1, 2 and 3 from the gut – and I think only sequenced 3 and 1. ILC2 are the dominant population in blood. Is it possible that the greater apparent overlap between the blood and lung relates to the type of ILCs sequenced rather than the biological overlap between these compartments?. More generally, does this analysis add useful information to the current study? Analysis of the transcriptional activity of ILCs during COVID may have provided evidence of their role in disease tolerance, as hypothesised by the authors. However, as the PBMC sequenced were uninfected healthy controls, this is impossible.

– Figure 2.B – it might be informative to plot males and females separately in each age bin. We know there is an equal ratio of males and females but we don’t know if this is equally distributed by age. From Figure 3 it looks like there may be a bias toward younger female and older male controls? Presenting it this way will make it easy for the reader to assess.

# Reply: Author response

**DOI:** 10.7554/eLife.74681.sa2

Essential Revisions (for the authors):

– The Introduction is too long and it is based on references of the past before the arrival of COVID-19.

To address these points, the Introduction was edited to start with a brief but more general statement about the features of severe COVID-19 (page 3, lines 89-92) and to include the two references suggested in comment #2 (PMID 32320677 and 34678611). Several sentences on innate lymphoid cells were deleted to focus the Introduction on COVID-19.

– One main concern is that the authors suggest that depletion of innate lymphoid cells (ILC) is the main driver of the lymphopenia of severe COVID-19. To prove this the authors need to provide the absolute counts of all subpopulations and to provide serial follow-up data. They also need to explain how hypoglobulinemia of patients is explained if depletion involves only ILC. There are at least two publications (PMID 32320677 and 34678611) suggesting defect in antigen presentation and they are not even referenced in this submission.

We thank the reviewers for the chance to clarify these points.

– It was not our intention to suggest that ILC defects are the primary cause of immune abnormalities such as lymphopenia or the defects in antigen presentation that the reviewer mentioned. As we wrote (page 6, lines 157-161), “The goal of this study was to determine whether the abundance of any blood lymphoid cell population was altered in COVID-19, independent of age, sex, and global lymphopenia, and whether abundance of any lymphoid cell population correlated with clinical outcome in SARS-CoV-2 infection.” After taking into account the effects of age and sex, our main finding was that reduction in ILC number was significantly associated with COVID-19 severity, risk and duration of hospitalization, and with increase in blood inflammatory markers such as CRP.

– To clarify this point we modified our abstract to say that our study “suggests” that lower ILC abundance, “contributes” to increased COVID-19 severity with age and in males (page 2, line 76).

– As stated above in response to point #1, we have expanded our introduction about the abnormalities associated with severe COVID-19 and have incorporated the two suggested references (page 3, lines 89-92).

– The authors need to provide data on the cytokine production capacity of ILC.

In response to this comment we have added a new panel showing that, in addition to being decreased in abundance, blood ILCs in people hospitalized for COVID-19 have significantly decreased capacity to produce amphiregulin (Figure 6D). The text of the abstract (page 2, line 69-71), results (page 44, lines 676-681), discussion (page 49, lines 786-788), and figure legends (page 46, line 703-706), have been modified accordingly.

– The authors should clarify when exactly blood sampling took place (at admission? During hospitalization? Before any treatment start?).

We now mention that samples from hospitalized patients were collected during admission (page 12, lines 165-168). In addition, we now mention that the COVID-19 blood samples were collected during the first wave of COVID-19, between March 31 and June 3^rd^, 2020 (page 12, lines 165-166); this time was before corticosteroids were demonstrated to be efficacious for severe COVID-19. Records of treatment interventions other than intubation were not available to us for these patients and this point has been added to the text (page 17, lines 271-272).

– According to Table 1 median duration of symptoms is about 20 days which is quiet a long period; normally patients are admitted after the first week. If sampling is not done at admission, administered therapy especially corticosteroids may influence flow cytometry results. If therapy was administered this has to be part of the regression results in Table 3.

We agree that timing of corticosteroids would be an important variable to include in Table 3. As discussed in response to point #4, this information was not available, and could not be included in the analysis. That being said, as indicated in the text (page 30, lines 457-463) and in the footnote to Table 3 (page 30), we included symptom duration at the time of sample collection as a covariate in the regression analysis presented in Table 3.

– Another concern is the absence in presentation of comorbidities as they can perhaps also influence results. If these data are unavailable, this is for sure a limitation and should be discussed.

In response to this suggestion we obtained information about diabetes mellitus. These data are now listed in Table 1 (page 18), mentioned in the text (page 17, lines 276-278), and included in the calculations for the odds of hospitalization presented Table 3 (page 30) and discussed on page 30 (lines 457-463). Accounting for diabetes mellitus diagnosis had minimal effect on the statistical results.

– In SARS-CoV2 infected adults, as reported widely, infections were highest in young adults, and mortality was highest in the elderly and increased approximately 1 log for each 20 year age group. Linear regression revealed that age, sex, and ILC and NK cell frequency were inversely correlated with hospitalization, and the association between ILC and NK frequency was retained when age and sex were adjusted for. Subsequent multiple logistic regression analysis showed that adjusting for age, sex, and symptom duration, only ILC frequency was significantly associated with an increased risk of hospitalization, the duration of hospital stay, and an increase in CRP. The same association between ILC frequency, but not other lymphocytes, and COVID was observed in an additional paediatric cohort. However, in rare Paediatric cases who developed MIS-C, both ILCs and T cells were significantly reduced. To link blood ILCs to what is happening on the lung, bioinformatic analysis of sequence data generated from sorted blood ILCS in compared to that of published gut and lung ILC datasets. Although, the authors argue this shows blood ILCs are transcriptionally more closely aligned to lung ILCs, the validity of this analysis is hard to judge without more detail and, as the data does not come from COVID subjects, its value in supporting the manuscript is unclear. Finally, ILCs from uninfected males produced less amphiregulin, important in lung homeostasis and repair.

We thank the reviewer for the extensive summary of our findings. The comment they make here is addressed in point #12 below.

– The loss of ILCs from circulation has been shown in several diseases, including HIV, TB, and COVID, as has the association between circulating ILCs and age. The strength of this manuscript is in using multiple regression to show that the association between ILC loss and disease severity is retained when age is controlled for, as age is such an important factor in COVID. However, the overall conclusion that elevated circulating ILCs support disease tolerance, while interesting, is not directly supported by any data. For example, a reduction in blood ILCs may indicate the recruitment of these cells to the lung, as has been shown for TB (Ardain et al., Nature 2019). In which case, an increased frequency of blood ILCs may not be protective per se, but just reflective of less lung involvement. Therefore, I suggest the title and abstract be altered to reflect the data presented more directly.

In response to this comment we have added new data and changed the text as recommended:

– The term “disease tolerance” has been removed from the title (page 1, line 3).

– The abstract now mentions that blood ILCs from people hospitalized with COVID-19 have significantly decreased capacity to produce amphiregulin (page 2, lines 69-71). See point number 3 above regarding new Figure 6D.

– The possibility that ILCs are sequestered in the lung is now mentioned in the text (page 40, lines 614-615).

– The study consistently refers to cell abundance but is actually reporting the frequency of cells. It is an important distinction in this context, as the total lymphocyte count declines with age. Therefore, if there are differences in the rates of decline, it can create the impression of an increase in specific subsets which are actually declining but just at a slower than the median (as may be happening with NK cells for example). To avoid any potential confusion, I suggest the authors present and discuss the data as frequencies of total lymphocytes (or PBMC as appropriate).

As suggested by the reviewer, the majority of the data are reported as ILCs per million lymphocytes. This is stated in the text and figure legends, and indicated in the Y axis labels on the figures. As the reviewer also points out, certain analyses were per million PBMCs; critical findings such as the effect of COVID-19 on ILCs were essentially the same per million lymphocytes or PBMCs (page 27, lines 418-420; Supplementary file 2e). The same was true for the other lymphoid cell subsets, including the significant increase in NK cells with age that was referred to by the reviewer.

– For the lymphocyte specific analysis, what is lacking is reporting of the other lymphocyte subsets, such as B-cells. It may not be possible to represent these as individual populations, depending on the markers used, but they could be presented as the remainder of the whole. In this way, the reader can get a sense of what is happening in the lymphocyte compartment as a whole. This is important given that absolute values are not available.

The reviewer makes an important point. To provide a sense of the lymphocyte compartment as a whole, we had analyzed lymphocyte abundance as a fraction of total PBMCs. This was described in the text on page 25, line 379-384,

“the effect of COVID-19 on total lymphocyte abundance was addressed with multiple linear regression. After accounting for effects of age and sex, individuals hospitalized with severe COVID-19 had 1.33-fold (95%CI: 1.49–1.19; p = 1.22 x 10^-6^) fewer total lymphocytes among PBMCs than did controls (Supplementary file 2d)”.

-I am a bit concerned that the synchronization of the paeds sample is too big an issue in this small sample size to give much confidence in the effects observed – the MISC subjects are measured at about 2months and 6-7 months, and the COVID at 6-8 months and one at 11 months. Given this, how much weight do the authors think this analysis carries?

We thank the reviewer for raising this question regarding the difference in timing of follow-up blood collections for the MISC-C subjects vs the COVID-19 subjects in Figure 4E (page 38). If the results had been different, the difference in timing would have been a problem. But, given the directionality of the effect we observed, this difference in timing highlights the divergence in rate of recovery of ILC numbers between these two groups: ILC abundance rebounded by 2 months of follow-up for the MIS-C patients, whereas ILC abundance had still not rebounded after 9 months of follow-up for the COVID-19 patients. We now emphasize this point in the text (page 36, lines 570-572).

– The authors compare the RNA read-out of the peripheral ILCs and they use previous arrays from lung ILCs to suggest that they are coming from the lung. This is far too arbitrary since this is a different cohort. Ardain et al., sorted ILC2s and ILC3s from the lung whilst Yudanin sorted ILC1, 2 and 3 from the gut – and I think only sequenced 3 and 1. ILC2 are the dominant population in blood. Is it possible that the greater apparent overlap between the blood and lung relates to the type of ILCs sequenced rather than the biological overlap between these compartments?. More generally, does this analysis add useful information to the current study? Analysis of the transcriptional activity of ILCs during COVID may have provided evidence of their role in disease tolerance, as hypothesised by the authors. However, as the PBMC sequenced were uninfected healthy controls, this is impossible.

Several changes were made to the manuscript in response to these comments:

– We agree that it would have been better if we had presented RNA-Seq data on lung ILCs from people hospitalized with COVID-19. The text now states that our attempts to profile ILCs from COVID-19 lung samples were unsuccessful (page 40, lines 610-612). In our previous work concerning intestinal ILCs from people living with HIV-1, biopsies had to be processed fresh within the hour (Nat Immunol 21:274); we were unable to profile intestinal ILCs from frozen or fixed tissues and suspect a similar issue holds for the viability of lung ILCs in COVID-19.

– We also agree that it would have been better if we had RNA-Seq data for ILCs isolated from the blood of people hospitalized with COVID-19. This was not possible because the limited sample available was prioritized for the FACS experiments. These samples had low numbers of ILCs which increased the difficulty of sorting viable cells from this population that is already rare in the blood of uninfected people.

– Given the significant reductions in blood ILCs in our study, and our inability to profile these rare cells from people with severe COVID, we felt it was important to provide some additional characterization of blood ILCs, even if only from uninfected individuals. Our analysis of global gene expression in blood ILCs from 9 donors shows close similarity to the expression profile of ILCs from the lung. While the reviewer is correct that this is driven in part by the ILC2 character of these cells, many of the 355 genes that distinguish blood and lung ILCs from ILCs of other tissues are not obvious ILC2 genes and could indicate other biologically relevant features in common between blood and lung ILCs (Figure 5B and C, page 42).

– The transcriptional analysis guided our phenotypic analysis of the blood ILCs (Figure 6 page 45), and, in the revised manuscript, we now show that blood ILCs in people hospitalized for COVID-19 have significantly decreased capacity to produce amphiregulin (Figure 6D). The text of the abstract (page 2, line 69-71), results (page 44, lines 676-681), discussion (page 49, lines 786-788), and figure legends (page 46, line 703-706), have been modified accordingly.

– Figure 2.B – it might be informative to plot males and females separately in each age bin. We know there is an equal ratio of males and females but we don't know if this is equally distributed by age. From Figure 3 it looks like there may be a bias toward younger female and older male controls? Presenting it this way will make it easy for the reader to assess.

To address the reviewer’s question about our control group we compared the male/female ratio for each age group against each of the other age groups in Figure 2. There were no significant differences in this ratio among any of the groups. A table showing this analysis has been added to the supplement (Supplementary file 2c).
